# Regulation of Flagellum Biosynthesis in Response to Cell Envelope Stress in *Salmonella enterica* Serovar Typhimurium

**DOI:** 10.1128/mBio.00736-17

**Published:** 2018-05-01

**Authors:** Imke Spöring, Sebastian Felgner, Matthias Preuße, Denitsa Eckweiler, Manfred Rohde, Susanne Häussler, Siegfried Weiss, Marc Erhardt

**Affiliations:** a Humboldt-Universität zu Berlin, Institute for Biology Bacterial Physiology, Berlin, Germany; b Junior Research Group Infection Biology of *Salmonella*, Helmholtz Centre for Infection Research, Braunschweig, Germany; c Department of Molecular Immunology, Helmholtz Centre for Infection Research, Braunschweig, Germany; d Department of Molecular Bacteriology, Helmholtz Centre for Infection Research, Braunschweig, Germany; e Central Facility for Microscopy, Helmholtz Centre for Infection Research, Braunschweig, Germany; f Institute of Immunology, Medical School Hannover, Hannover, Germany; Ohio State University; Max Planck Institute for Infection Biology

## Abstract

Flagellum-driven motility of Salmonella enterica serovar Typhimurium facilitates host colonization. However, the large extracellular flagellum is also a prime target for the immune system. As consequence, expression of flagella is bistable within a population of *Salmonella*, resulting in flagellated and nonflagellated subpopulations. This allows the bacteria to maximize fitness in hostile environments. The degenerate EAL domain protein RflP (formerly YdiV) is responsible for the bistable expression of flagella by directing the flagellar master regulatory complex FlhD_4_C_2_ with respect to proteolytic degradation. Information concerning the environmental cues controlling expression of *rflP* and thus about the bistable flagellar biosynthesis remains ambiguous. Here, we demonstrated that RflP responds to cell envelope stress and alterations of outer membrane integrity. Lipopolysaccharide (LPS) truncation mutants of *Salmonella* Typhimurium exhibited increasing motility defects due to downregulation of flagellar gene expression. Transposon mutagenesis and genetic profiling revealed that σ^24^ (RpoE) and Rcs phosphorelay-dependent cell envelope stress response systems sense modifications of the lipopolysaccaride, low pH, and activity of the complement system. This subsequently results in activation of RflP expression and degradation of FlhD_4_C_2_ via ClpXP. We speculate that the presence of diverse hostile environments inside the host might result in cell envelope damage and would thus trigger the repression of resource-costly and immunogenic flagellum biosynthesis via activation of the cell envelope stress response.

## IMPORTANCE

Pathogenic bacteria such as *Salmonella* Typhimurium sense and adapt to a multitude of changing and stressful environments during host infection. At the initial stage of gastrointestinal colonization, *Salmonella* uses flagellum-mediated motility to reach preferred sites of infection. However, the flagellum also constitutes a prime target for the host’s immune response. Accordingly, the pathogen needs to determine the spatiotemporal stage of infection and control flagellar biosynthesis in a robust manner. We found that *Salmonella* uses signals from cell envelope stress-sensing systems to turn off production of flagella. We speculate that downregulation of flagellum synthesis after cell envelope damage in hostile environments aids survival of *Salmonella* during late stages of infection and provides a means to escape recognition by the immune system.

## INTRODUCTION

The enteropathogen Salmonella enterica serovar Typhimurium is able to move in a directed manner using flagellum-mediated propulsion in a process known as chemotaxis (1). The bacterial flagellum is a complex macromolecular machine and made via self-assembly of dozens of different proteins (2, 3). A mature external filament consists of ~20,000 flagellin subunits; thus, *de novo* production of flagella constitutes a high metabolic burden (4). Accordingly, flagellar biosynthesis is tightly controlled at both the transcriptional and posttranslational levels. At the transcriptional level, flagellar gene expression is hierarchically organized. The *flhDC* master regulatory operon is under the control of a class 1 promoter and is activated in response to a plethora of environmental signals (5). The master regulatory complex FlhD_4_C_2_ activates gene expression from class 2 promoters. Its gene products build the basal body and the hook of the flagellum. Once the hook-basal-body (HBB) complex is completed, expression of genes under the control of class 3 promoters, including genes coding for the filament, motor-force generators, and chemosensory system, is derepressed (3, 6). Transcriptional regulation of the flagellar master regulator is a complex process and is controlled by numerous global transcriptional regulators (e.g., RcsB, RflM, HilD, and LrhA), which act on the level of the class 1 promoter of *flhDC* (7–10). Posttranslational regulation of FlhD_4_C_2_ is dependent on the activity of degenerate EAL domain-containing protein RflP (regulator of FlhDC proteolysis; formerly known as YdiV [11]). It targets FlhD_4_C_2_ protein complexes with respect to proteolytic degradation by the ClpXP protease (12). In *S*. Typhimurium, the concentration of nutrients tunes the fractions of motile and nonmotile bacteria. RflP was shown to be responsible for the observed nutrient-dependent phenotypic heterogeneity of flagellar gene expression (13–15).

The ability to move is an important virulence trait of *Salmonella*. However, the flagellar filament is also highly immunogenic. Thus, flagellum synthesis is suppressed at the primary site of infection to avoid immune recognition by either Toll-like receptor 5 (TLR-5) or the NRLC4/NAIP5/6 inflammasome (16, 17). Further, *S*. Typhimurium appears to exploit the above-mentioned heterogeneous regulation of flagellum production during the early stages of infection in addition to population-wide repression of flagellar gene expression during systemic infection. The bistable regulation of flagellum synthesis may prime subpopulations of *Salmonella* to both evade the immune system in deeper organs and survive in the small intestine, where motility might be advantageous (18, 19). However, knowledge concerning the signals controlling the expression of the bistable switch protein RflP remained elusive.

S. Typhimurium employs sophisticated sensory signal transduction systems to sense and adapt to its environment. Throughout host infection, *Salmonella* encounters various stress conditions, including temperature and oxidative/nitrosative or cell envelope stress, within a short period of time. These diverse environments require important lifestyle decisions by the bacteria. In order to sense external stresses, *Salmonella* relies on several sensory systems. This allows the bacteria to respond to various environments and to fine-tune its gene expression profile. In particular, the Psp, Bae, Cpx, and Rcs systems as well as the Rse-σ^24^ system are known to sense and drive gene expression in response to cell envelope stress (20, 21). Signals that trigger the σ^24^ response include misfolded proteins located in the periplasm. However, the σ^24^ regulon also comprises proteins connected to lipopolysaccharide (LPS) synthesis (21). In addition, a drastic reduction of the LPS moieties results in perturbations of the outer membrane (OM) (22).

Recently, we characterized novel factors affecting motility of *S*. Typhimurium (23). We observed strongly reduced motility of a deep rough LPS mutant (Δ*rfaG*) deficient in outer core and O-antigen synthesis. Similar findings were also reported for mutant Δ*rfaD*, another deep rough LPS mutant of *Salmonella*, which is also unable to synthesize the inner core (22, 24), as well as for several Escherichia coli LPS mutants (25). In E. coli, LPS truncation mutants were shown to display reduced flagellin levels due to a defect in *flhDC* transcription (25, 26). The motility phenotype was rescued by deletion of *rcsB*, *rcsD*, or *rcsF*. Activation of the Rcs phosphorelay system has been shown to repress *flhDC* expression (27, 28), suggesting that repression of motility occurs via the Rcs signaling pathway in E. coli mutants defective in LPS synthesis.

In the present study, we aimed to characterize the regulatory link between LPS truncations and abrogated motility in *S*. Typhimurium. To address this issue, we characterized *Salmonella* LPS mutants Δ*rfaL*, Δ*rfaG*, and Δ*rfaD* and observed that decreases in flagellum expression were dependent on the length of the LPS structure; i.e., increasing truncations of the LPS resulted in stronger repression of flagellar synthesis. A Tn*5*-based transposon mutagenesis approach and transcriptional profiling revealed that activation of cell envelope stress sensing systems, including stress sigma factor σ^24^ and the Rcs system, results in repression of flagellar motility via the action of RflP. Expression of *rflP* was induced in response to various forms of cell envelope stress, including truncations of the LPS structure, EDTA, acidic pH, or serum (i.e., the complement system). We thus propose that *Salmonella* senses alterations in cell envelope integrity in response to host defense mechanisms and subsequently downregulates flagellar synthesis to maximize its fitness in hostile environments inside the host.

## RESULTS

### Sequential truncation of the LPS results in stepwise downregulation of flagellum synthesis.

**FIG 1:**
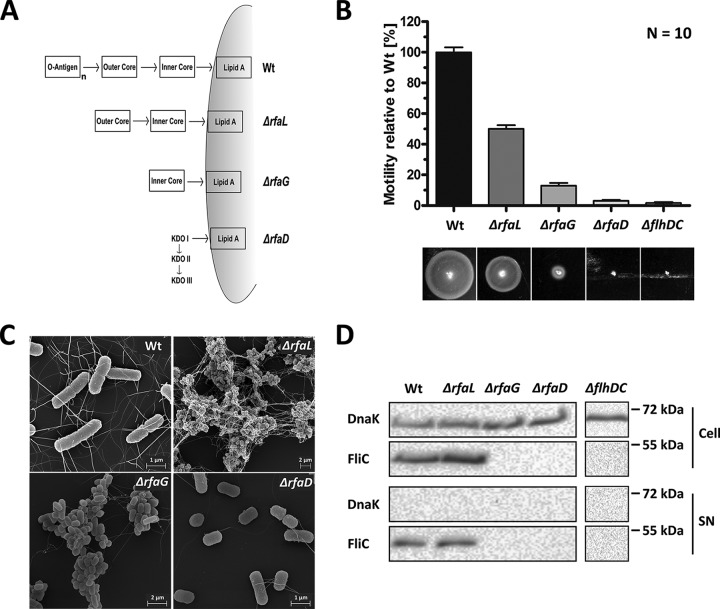
Phenotypic characterization of *Salmonella* LPS mutants regarding motility and flagellation. (A) Schematic representation of LPS structure. Genes encoding the enzymes for particular steps in LPS synthesis were deleted, resulting in the depicted LPS phenotype. (Adapted from reference 56 with permission of the publisher.) (B) Swimming motility of Wt and LPS mutant bacteria assessed on semisolid agar after 4 h of incubation at 37°C. The Δ*flhDC* mutant strain served as a negative control. Bars represent means + standard errors of the means of results from 2 individual experiments (*n* = 10). (C) Scanning electron microscopy of Wt *Salmonella* and the LPS Δ*rfaL*, Δ*rfaG*, and Δ*rfaD* mutants. (D) Western blot of FliC protein levels of Wt and LPS mutants in whole-cell extract (FliC expression; upper panel) and supernatant (SN) (FliC secretion; lower panel). Protein levels were monitored by SDS-PAGE and immunoblotting. The protein DnaK served as an intracellular control.

Motility is impaired in deep rough LPS truncation mutants Δ*rfaG* and Δ*rfaD* (23, 24). We thus analyzed the motility phenotype of another rough LPS *Salmonella* mutant deficient for *rfaL*. The Δ*rfaL* mutant lacks the O-antigen, while the Δ*rfaG* mutant lacks the outer core and the O-antigen. The Δ*rfaD* mutant retains only the KDO segment of the outer LPS structure (Fig. 1A). Increasing truncations of the LPS structure resulted in decreasing motility (Fig. 1B), which confirmed previous findings in E. coli (25).

Scanning electron microscopy revealed that wild-type (Wt) *Salmonella* bacteria were fully flagellated. Similarly, the *rfaL*-deficient strain was flagellated. Its reduced motility may arise from strong aggregation of bacterial cell bodies (Fig. 1C). In contrast, the Δ*rfaG* mutant expressed significantly lower levels of flagella, which explains the strongly reduced motility. Finally, complete removal of the LPS core structure (Δ*rfaD*) entirely abrogated flagellation and motility (Fig. 1B and C; see also Fig. S1 in the supplemental material). This was further confirmed by the absence of flagellin (FliC) production and secretion (Fig. 1D).

10.1128/mBio.00736-17.1 FIG S1  Swarming motility of the *Salmonella* LPS mutants. Swarming motility was analyzed on plates containing 0.6% agar after 8 h of incubation at 37°C. Bars represent means + standard errors of the means of results from 2 individual experiments (*n* = 14). Download FIG S1, PDF file, 0.03 MB. Copyright © 2018 Spöring et al. 2018 Spöring et al. This content is distributed under the terms of the Creative Commons Attribution 4.0 International license.

**FIG 2:**
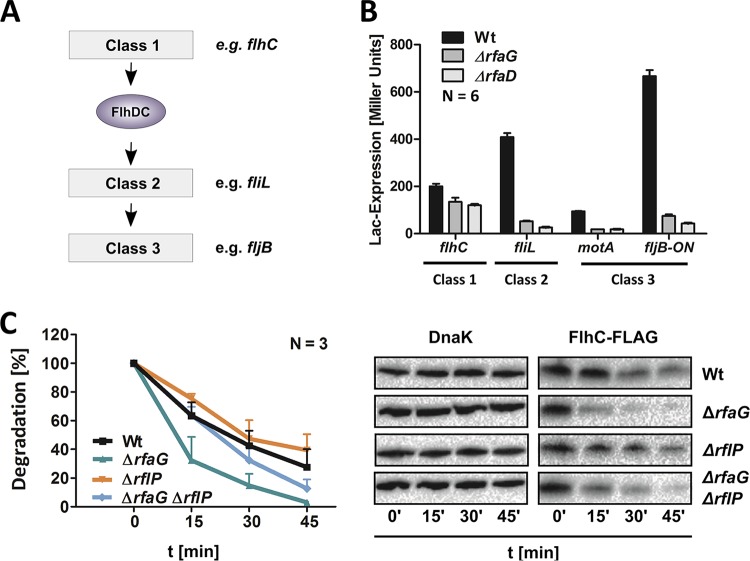
Analysis of flagellar gene expression in the LPS mutants. (A) Schematic of the hierarchical flagellar gene regulation cascade. The FlhDC flagellar master regulator complex is transcribed from a class 1 promoter. FlhD_4_C_2_ induces expression of class 2 promoter genes (e.g., *fliL*). After completion of the flagellar hook basal body complex, transcription of class 3 promoter genes (e.g., *fljB*) commences. (B) Relative *flhC* (class 1), *flgE* (class 2), and *fliC* (class 3) gene expression levels of the LPS mutant strains Δ*rfaL*, Δ*rfaG*, and Δ*rfaD* compared to Wt *Salmonella*. Bars represent means + standard errors of the means of results from 2 individual experiments (*n* = 6). (C) Degradation assay of FlhC-FLAG protein levels over 60 min. Synthesis was stopped by treatment with spectinomycin and chloramphenicol. A Western blot of FlhC-FLAG protein levels of Wt and mutants in whole-cell extract is shown. Protein levels were monitored by SDS-PAGE and immunoblotting. The protein DnaK served as an intracellular control.

We next investigated whether the loss of flagella was caused by alterations of the membrane due to the LPS truncations or was due to regulatory effects on flagellar gene expression as previously suggested for E. coli (25). Therefore, we tested gene expression from the three flagellar promoter classes in the Wt, Δ*rfaG*, and Δ*rfaD* strains using transcriptional *lacZ* reporter fusions (Fig. 2A). Flagellar gene expression from class 2 (*fliL*) and class 3 (*motA* and *fljB*) promoters was strongly reduced in the LPS truncation mutants. Gene expression from the class 1 promoter (*flhC*), however, was affected only slightly (Fig. 2B). The pronounced downregulation of flagellar transcripts expressed from class 2 and class 3 promoters was confirmed by reverse transcription and quantitative real-time PCR (RT-qPCR) (Fig. S2). These findings suggested the occurrence of primarily posttranscriptional regulation at the level of the FlhD_4_C_2_ master regulatory complex. The FlhD_4_C_2_ protein complex is known to be subject to proteolytic degradation (12). Accordingly, we tested the stability of epitope-tagged FlhC protein after arrest of *de novo* protein synthesis and found that the FlhC protein was degraded substantially faster in the Δ*rfaG* LPS truncation mutant than in the Wt strain (Fig. 2C). These results show that the reduced motility of the LPS truncation mutants was due to posttranscriptional regulation at the level of the flagellar master regulatory complex, FlhD_4_C_2_.

10.1128/mBio.00736-17.2 FIG S2  Relative gene expression levels of the flagellar genes in *Salmonella* LPS mutants. Relative *flhC* (class 1), *flgE* (class 2), and *fliC* (class 3) gene expression levels of LPS mutant strains Δ*rfaL*, Δ*rfaG*, and Δ*rfaD* compared to Wt *Salmonella* were analyzed by RT-qPCR. Bars represent means + standard errors of the means of results from 2 individual experiments (*n* = 4). *, *P* ≤ 0.05; **, *P* ≤ 0.01. Download FIG S2, PDF file, 0.2 MB. Copyright © 2018 Spöring et al. 2018 Spöring et al. This content is distributed under the terms of the Creative Commons Attribution 4.0 International license.

### Induction of cell envelope stress using EDTA mimics an LPS truncation phenotype.

**FIG 3:**
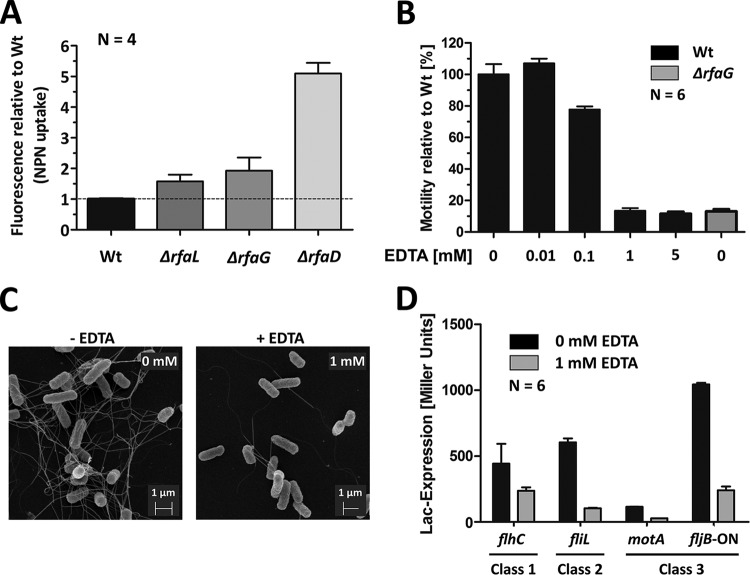
The motility defect of LPS mutants can be mimicked by EDTA supplementation. (A) Change in NPN uptake as an indicator of outer membrane instability in the Δ*rfaL*, Δ*rfaG*, and Δ*rfaD* LPS mutants. Bars represent means + standard errors of the means of results from 1 individual experiment (*n* = 4). (B) The swimming motility of Wt bacteria was assessed on semisolid agar supplemented with 0, 0.01, 0.1, 1, and 5 mM EDTA and compared to that of Δ*rfaG* LPS mutant bacteria after 4 h of incubation at 37°C. Bars represent means + standard errors of the means of results from 2 individual experiments (*n* = 6). (C) Scanning electron microscopy of Wt *Salmonella* bacteria grown overnight in LB or in LB supplemented with 1 mM EDTA. (D) Relative *flhC* (class 1), *flgE* (class 2), and *fliC* (class 3) gene expression levels of the Wt *Salmonella* bacteria. The bacteria were grown for 1.5 h in LB. A null experiment was performed or 1 mM EDTA was added, and bacteria were cultured for ~1.5 h prior to gene expression measurement. Bars represent means + standard errors of the means of results from 2 individual experiments (*n* = 6).

Modifications of the LPS moiety result in perturbations of the outer membrane (OM) (22). We thus hypothesized that the observed reduction of flagellar gene expression in the LPS truncation mutants was indirect, since perturbations of the outer membrane might result in general cell envelope stress. To test this hypothesis, we measured destabilization of the OM in LPS mutants using the established 1-N-phenylnaphtylamine (NPN) uptake assay (29, 30). NPN is an environment-sensitive dye that emits fluorescence when integrated into membranes but not in solution. When the OM is compromised, the dye is able to enter and integrate into the inner membrane. In line with our hypothesis, the levels of fluorescence intensity of the Δ*rfaG* and Δ*rfaD* LPS mutants were increased by 1.6-fold and 4.3-fold compared to the Wt, respectively (Fig. 3A).

We next used EDTA to mimic cell envelope stress independently of truncations of the LPS as described before (31). Wt *Salmonella* incubated with 1 mM EDTA exhibited a phenotype in terms of flagellation and motility similar to that seen with an *rfaG*-deficient strain (Fig. 3B to D). This observation was consistent with our hypothesis that flagellum synthesis is downregulated by an apparent cell envelope stress induced by either LPS truncations or perturbations of the outer membrane after addition of EDTA.

### RflP is a key regulator that controls flagellum expression during envelope stress.

**FIG 4:**
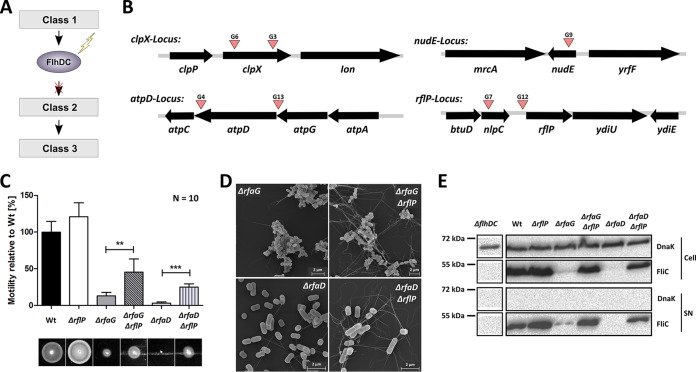
Analysis of flagellar gene expression in the LPS mutants. (A) Schematic model of the stress-mediated downregulation of motility and assumption for design of the transposon screen. The screen is based on the use of the *fliL*::*lac* (class 2) reporter gene to screen for mutants that reestablish class 2 gene expression in a Δ*rfaG* mutant. (B) Schematic depiction of transposon insertion sites found in the screen. (C) Swimming motility of the Wt strain, a Δ*rflP* mutant, and Δ*rfaG* and Δ*rfaD* LPS mutants in the absence and presence of *rflP* assessed on semisolid agar after 4 h of incubation at 37°C. Bars represent means + standard errors of the means of results from 2 individual experiments (*n* = 10). (D) Scanning electron microscopy of LPS mutant strains Δ*rfaG* and Δ*rfaD* in the absence and presence of *rflP*. (E) Western blot of FliC protein levels of Wt and LPS mutant strains Δ*rfaG* and Δ*rfaD* in the absence or presence of *rflP* in whole-cell extract (FliC expression; upper panel) and supernatant (SN) (FliC secretion; lower panel). Protein levels were monitored by SDS-PAGE and immunoblotting. The protein DnaK served as an intracellular control. **, *P* ≤ 0.01; ***, *P* ≤ 0.001.

To elucidate the regulatory circuit underlying the observed motility and flagellum biosynthesis defect (Fig. 4A), we performed an unbiased random Tn*5* transposon mutagenesis screen. We generated a Δ*rfaG Salmonella* strain that harbored a *fliL-lacZ* transcriptional fusion to report flagellar gene expression from a class 2 promoter. On lactose (Lac) indicator media, a Δ*rfaG* mutant displayed a Lac-negative (Lac^−^) phenotype, whereas the Wt strain was Lac^+^. This reporter strain allowed us to screen a pool of Tn*5* insertion mutants for restored flagellar class 2 gene expression in the Δ*rfaG* mutant. A nonsaturating screen of approximately 9,000 Tn*5* insertions identified seven transposon insertions that displayed a Lac^*+*^ phenotype. Four Lac^+^ transposon insertions were found in close proximity to the *rflP* and *clpX* loci (Fig. 4B; see also Table S1 in the supplemental material). RflP and ClpXP are known factors involved in posttranscriptional regulation of FlhD_4_C_2_ (12, 13). From these results, we hypothesized that a signal reporting cell envelope stress would activate RflP and would thereby cause proteolytic degradation of FlhD_4_C_2_.

10.1128/mBio.00736-17.6 TABLE S1  Chromosomal EZ-Tn*5* transposon integration sites. Download TABLE S1, DOCX file, 0.05 MB. Copyright © 2018 Spöring et al. 2018 Spöring et al. This content is distributed under the terms of the Creative Commons Attribution 4.0 International license.

We therefore deleted the *rflP* gene to confirm the role of RflP as the key regulator responsible for downregulation of flagellar class 2 gene expression under cell envelope stress conditions. Consistent with our hypothesis, motility, flagellum synthesis, and expression of the flagellin FliC were partially restored in the double deletion mutant Δ*rfaG* Δ*rflP* (Fig. 4C to E). In accordance with our hypothesis, the stability of epitope-tagged FlhC protein after arrest of *de novo* protein synthesis was increased in a Δ*rfaG* Δ*rflP* double mutant compared to the Δ*rfaG* single mutant (Fig. 2C). Importantly, motility was restored to a similar extent in a Δ*rfaG* Δ*clpX* double mutant (Fig. S3A and B). An increase in flagellum synthesis was also observed for double mutant Δ*rfaL* Δ*rflP*. However, aggregation of bacterial cell bodies prevented a quantitative analysis of the motility phenotype (Fig. S3C and D).

10.1128/mBio.00736-17.3 FIG S3  Deletion of *clpX* and *atpA* partially restores motility and flagellation of LPS mutants, and deletion of *rflP* modulates flagellation of Δ*rfaL* mutant bacteria without affecting motility. (A) Scanning electron microscopy of LPS mutant strain Δ*rfaG* in the absence of *atpA* (left) and *clpX* (right). (B) Swimming motility of the Wt strain, a Δ*clpX* mutant, and LPS mutant Δ*rfaG* in the absence and presence of *clpX* assessed on semisolid agar after 4 h of incubation at 37°C. Bars represent means + standard errors of the means of results from 2 individual experiments (*n* = 6). (C) Scanning electron microscopy of the Δ*rfaL* mutant and the Δ*rfaL ΔrflP* mutant. (D) Swimming motility of the Wt strain and the Δ*rfaL* mutant strain in the absence and presence of *rflP* assessed on semisolid agar after 4 h of incubation at 37°C. Bars represent means + standard errors of the means of results from 2 individual experiments (*n* = 6). (E) Relative *rflP* gene expression levels of LPS mutant strains Δ*rfaL* and Δ*rfaL ΔrflP* compared to Wt *Salmonella* analyzed by RT-qPCR. Bars represent means + standard errors of the means of results from 2 individual experiments (*n* = 4). Download FIG S3, PDF file, 0.2 MB. Copyright © 2018 Spöring et al. 2018 Spöring et al. This content is distributed under the terms of the Creative Commons Attribution 4.0 International license.

**FIG 5:**
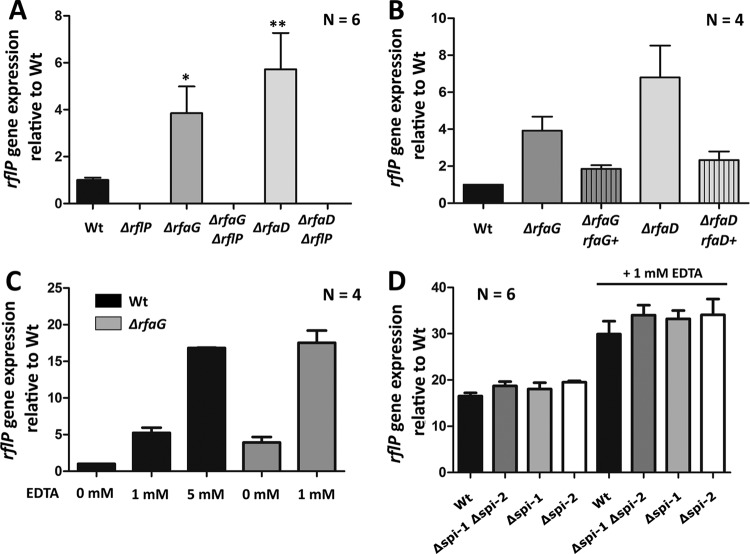
Analysis of *rflP* gene expression in the LPS mutants and upon EDTA addition. (A) Relative *rflP* gene expression levels analyzed by reverse transcription and quantitative real-time PCR (RT-qPCR) of LPS mutant strains Δ*rfaG* and Δ*rfaD* in the absence and presence of *rflP* compared to Wt *Salmonella*. Bars represent means + standard errors of the means of results from 3 individual experiments (*n* = 6). (B) Relative *rflP* gene expression levels analyzed by RT-qPCR of LPS mutant strains Δ*rfaG* and Δ*rfaD* and their complemented strains using the chromosomally integrated P_BAD_ system (Δ*rfaG rfaG* = Δ*araBAD*::*rfaG* Δ*rfaG*::*aph* [EM4410]; Δ*rfaD rfaD*+ = Δ*araBAD*::*rfaD* Δ*rfaD*::*aph* [EM4411]). Bars represent means + standard errors of the means of results from 2 individual experiments (*n* = 4). (C) Relative *rflP* gene expression determined by qRT-PCR in LPS mutant strain Δ*rfaG* compared to Wt *Salmonella*. The bacteria were grown for 1.5 h in LB. Null experiments were performed or 1 or 5 mM EDTA was added, and bacteria were cultured for ~1.5 h prior to gene expression measurement. Bars represent means + standard errors of the means of results from 2 individual experiments (*n* = 4). (D) *rflP-lac* fusion expression in mutant Δ*spi-1*, mutant Δ*spi-2*, and a Δ*spi-1* Δ*spi-2* double mutant with and without EDTA. The bacteria were grown for 1.5 h in LB. EDTA was added, and bacteria were cultured for ~1.5 h prior to gene expression measurement by β-galactosidase assay. Bars represent means + standard errors of the means of results from 2 individual experiments (*n* = 6). *, *P* ≤ 0.05; **, *P* ≤ 0.01.

As mentioned above, we speculated that cell envelope perturbations result in activation of *rflP* gene expression. Thus, we determined the transcript levels of *rflP* in the LPS truncation mutants using RT-qPCR. As expected, we detected increased *rflP* gene expression in the LPS truncation mutants which correlated with increased levels of cell envelope stress (Fig. 5A; see also Fig. S3E). Complementation of Δ*rfaG* or Δ*rfaD* mutants reduced *rflP* expression to Wt levels (Fig. 5B). Addition of 1 mM EDTA also increased transcriptional activation of *rflP* 4-fold to 5-fold compared to the untreated control results, and the level of activation resembled that of *rflP* expression in the Δ*rfaG* mutant (Fig. 5C).

EDTA and EGTA are known magnesium (Mg^2+^) and calcium (Ca^2+^) chelators, and depletion of Mg^2+^ induces *Salmonella* pathogenicity island 2 (SPI-2) virulence gene expression in *Salmonella* via the PhoP-PhoQ two-component system (TCS) (32). To test if enhanced virulence gene expression in response to low Mg^2+^ levels contributes to the observed upregulation of *rflP* upon addition of chelators, we tested *rflP* gene expression in mutants that lacked major *Salmonella* pathogenicity islands SPI-1 and SPI-2. When challenged with EDTA, *rflP* was upregulated in all tested strains to an extent similar to that determined for the Wt. From this, we concluded that activation of the PhoP-PhoQ system does not contribute to the observed motility defect under conditions of outer membrane perturbation (Fig. 5D).

**FIG 6:**
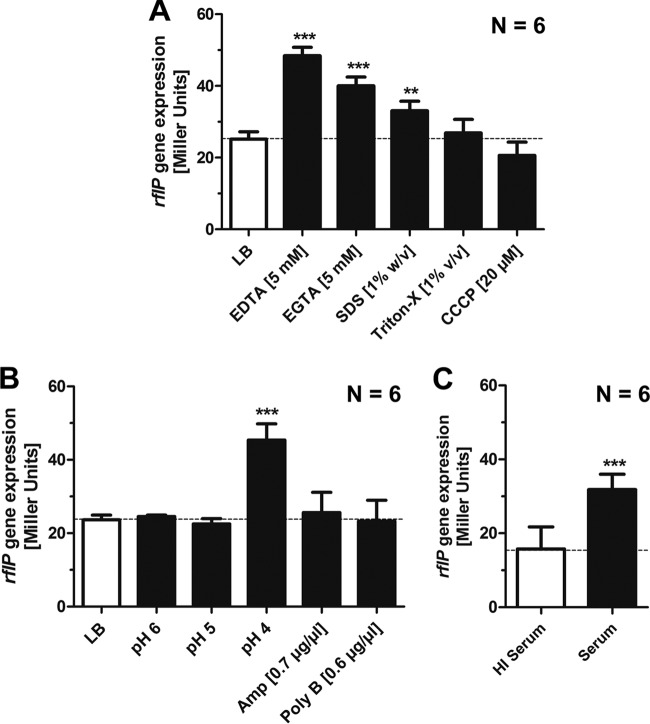
Analysis of *rflP* gene expression upon addition of various envelope stressing conditions. (A) Chemical membrane stressing agents: EDTA, EGTA, SDS, Triton, and CCCP. (B) Changes of the medium conditions: pH and the antibiotic ampicillin (Amp) and the antimicrobial peptide polymyxin B (Poly B). (C) Human serum containing the complement system. The bacteria were grown for 1.5 h in LB. The stressors were added, and bacteria were cultured for ~1.5 h prior to gene expression measurement by β-galactosidase assay. Bars represent means + standard errors of the means of results from 2 individual experiments (*n* ≥ 4). **, *P* ≤ 0.01; ***, *P* ≤ 0.001.

A variety of chemical and biological agents may induce cell envelope stress. Therefore, we challenged Wt *Salmonella* with cell envelope-acting compounds. These included EGTA, acidic pH, antimicrobials such as polymyxin B or ampicillin, detergents (Triton-X and SDS), and the ionophore carbonyl cyanide *m*-chlorophenyl hydrazone (CCCP). In addition, the effects of the complement system in human serum as part of the innate immune system were tested. Addition of EGTA, the complement system, and pH 4, but not addition of detergents, polymyxin B, ampicillin, and pH 5, affected *rflP* expression. This suggested that the activation of *rflP* expression responded to specific cell envelope stress pathways (Fig. 6).

### Transcriptome analysis revealed the involvement of σ^24^ in activation of *rflP* expression.

**FIG 7:**
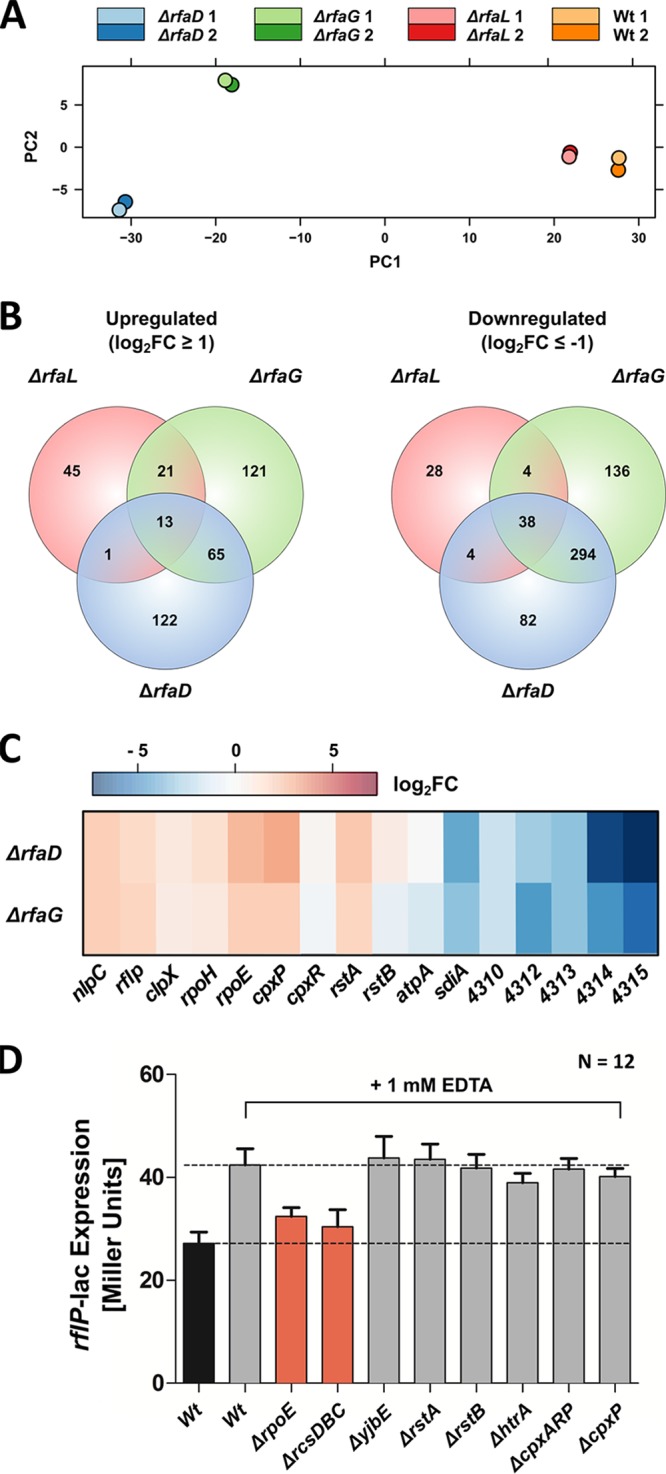
Transcriptome analyses of the LPS mutants reveals involvement of stress sigma factor σ^24^ in activation of *rflP*. (A) Principal-component analysis of the transcriptomes of LPS mutants Δ*rfaL*, Δ*rfaG*, and Δ*rfaD*. Colored circles depict the two biological replicates of each mutant. (B) Venn diagram of the LPS mutant transcriptomes. (C) Averaged log2-fold change (log_2_FC) of expression levels of selected genes of interest in the Δ*rfaG* and Δ*rfaD* mutants, including the following: (i) genes involved in transposon mutagenesis (*nlpC*, *rflP*, *clpX*, *atp* operon); (ii) genes encoding stress response sigma factors (*rpoH*, *rpoE*); (iii) genes encoding Rst (*rstAB*) and Cpx (*cpxPR*) signal transduction systems; (iv) genes corresponding to an unknown putative two-component system (STM4310-STM4315). (D) *rflP* expression in Δ*rpoE*, Δ*rcsBDC*, Δ*yjbE*, Δ*rstA*, Δ*rstB*, Δ*htrA*, Δ*cpxAR* Δ*cpxP* (Δ*cpxARP*), and Δ*cpxP* mutants. The LB medium was supplemented with 1 mM EDTA to induce *rflP* expression in response to cell envelope stress. *Salmonella* Wt bacteria grown in LB served as controls. The bacteria were grown for 1.5 h in LB. EDTA (1 mM) was added, and bacteria were cultured for ~1.5 h prior to gene expression measurement. Bars represent means + standard errors of the means of results from 2 individual experiments (*n* = 6).

We next analyzed the transcriptome of Δ*rfaL*, Δ*rfaG*, and Δ*rfaD* LPS truncation mutants to elucidate the regulatory link between RflP and cell envelope stress. Principal-component analysis indicated that the gene expression profiles of the Δ*rfaG* and Δ*rfaD* mutants were distinct from those of the Δ*rfaL* mutant and Wt *Salmonella* (Fig. 7A; see also Fig. S4). Differential gene expression analysis revealed 217 differentially regulated genes in the Δ*rfaL* mutant (136 upregulated and 81 downregulated), 716 in the Δ*rfaG* mutant (232 upregulated and 484 downregulated), and 619 in the Δ*rfaD* mutant (201 upregulated and 418 downregulated) in comparison to the Wt (Fig. 7B; see also Table S2). KEGG pathway analysis confirmed the downregulation of flagellar, chemotaxis, and fimbrial genes in the Δ*rfaG* and Δ*rfaD* mutants. Interestingly, the virulence-associated injectisome systems and effector proteins encoded on SPI-1 and SPI-2 were also strongly downregulated in *rfaG*-deficient and *rfaD-*deficient bacteria (Fig. S5). Further, analysis of differentially regulated genes showed downregulation of metabolic genes involved in the tricarboxylic acid (TCA) cycle and upregulation of tRNA synthesis genes. An operon encoding a putative signal transduction system (*S*. Typhimurium 4310 [STM4310] to STM4315) was strongly downregulated in the Δ*rfaD* and Δ*rfaG* mutants. Also, the *sdiA* quorum sensing regulator gene was profoundly downregulated (for mutant Δ*rfaG*, log_2_ fold change [log_2_FC] = −3.04; for mutant Δ*rfaD*, log_2_FC = −3.87). Components of the F_O_F_1_ ATPase, such as *atpA*, were downregulated in the LPS truncation mutants (for mutant Δ*rfaG*, log_2_FC = −1; for mutant Δ*rfaD*, log_2_FC = −1.9) (Fig. 7C). The idea of possible involvement of expression of the ATP synthase in cell envelope stress-mediated downregulation of flagella was supported by our Tn*5* mutagenesis results. Two Lac^+^ insertions were found disrupting *atpD*, the β-subunit of the F_O_F_1_ ATPase. However, how production of the ATPase affects flagellar gene expression under cell envelope stress conditions remains to be elucidated (Fig. 4B; see also Table S2).

10.1128/mBio.00736-17.4 FIG S4  Scatterplot of the mutant Δ*rfaL*, Δ*rfaG*, and Δ*rfaD* transcriptomes. Data from significantly and differentially regulated genes with Log_2_FC values of ≤1 and ≥1 are shown in red (upregulated) and blue (downregulated), respectively. Data from genes that were not significantly and differentially regulated are shown in gray. Prominent differentially regulated genes and gene clusters are indicated by boxes. Download FIG S4, PDF file, 0.4 MB. Copyright © 2018 Spöring et al. 2018 Spöring et al. This content is distributed under the terms of the Creative Commons Attribution 4.0 International license.

10.1128/mBio.00736-17.5 FIG S5  Growth curves of putative sensor mutants. Growth was monitored over time at 37°C of deletion mutants *cpxAR cpxP* (Δ*cpxARP*), *cpxP*, *nlpC*, *yjbE*, *rstA*, *rstB*, and *htrA* compared to that of the Wt strain. Curves represent means of results from 1 individual representative experiment (*n* = 8). Download FIG S5, PDF file, 0.2 MB. Copyright © 2018 Spöring et al. 2018 Spöring et al. This content is distributed under the terms of the Creative Commons Attribution 4.0 International license.

10.1128/mBio.00736-17.7 TABLE S2  *In vitro* transcriptome analysis of the genes in the LPS mutants that were significantly up- and downregulated compared to the Wt results. Download TABLE S2, DOCX file, 0.3 MB. Copyright © 2018 Spöring et al. 2018 Spöring et al. This content is distributed under the terms of the Creative Commons Attribution 4.0 International license.

Corroborating our previous results, *clpX* and *rflP* were upregulated in the LPS truncation mutants. Interestingly, *nlpC* transcription was also slightly enhanced in the mutants (for mutant Δ*rfaD*, log_2_FC = 1.7; for mutant Δ*rfaG*, log_2_FC = 1.9). The thus-far-uncharacterized NlpC lipoprotein is encoded directly upstream of *rflP*, and we isolated Lac^+^ insertions in *nlpC* in our Tn*5* transposon screen. Insertions in *nlpC* were previously found in another transposon screen, where RflP was shown to be upregulated under starvation conditions (13). These findings suggested a potential regulatory connection between NlpC and RflP.

We further found that RstA, the response regulator of the Rst two-component system (TCS), was upregulated in the LPS truncation mutants (for mutant Δ*rfaD*, log_2_FC = 1.91; for mutant Δ*rfaG*, log_2_FC = 1.64). Overexpression of RstA induces degradation of σ^38^ (RpoS) and alters biofilm formation, and the Rst TCS promotes expression of an iron transport protein, FeoB, under iron-replete conditions (33, 34). However, we found that *feoAB* and fimbriae, as important biofilm-associated factors, were downregulated in the transcriptome of the LPS truncation mutants.

We next analyzed the transcriptome with respect to known cell envelope stress sensing systems. The phage-shock-protein (Psp) response system is a regulatory system which senses cell envelope perturbations. The Psp system is activated by alterations of the proton motif force (pmf), which result in upregulation of the *pspABCDE* operon and *pspG* in a σ^54^-dependent manner (20, 21). However, our transcriptome analysis revealed downregulation of the phage-shock response components *pspABC* in the LPS truncation mutants (e.g., for *pspA*, Δ*rfaD* mutant log_2_FC = −1.74 and Δ*rfaG* mutant log_2_FC = −2.08) indicating that this pathway is not relevant in that context. Consistently, disruption of the pmf using the CCCP uncoupler did not increase *rflP* expression (Fig. 6A).

Another TCS that senses and responds to cell envelope stress is CpxRA. The Cpx response is activated by adhesion; pH stress; or aggregated, misfolded periplasmic proteins. Under stress conditions, the CpxR-P phosphorylated response regulator negatively regulates genes coding for the stress σ-factor σ^24^ (*rpoE*) and the related cell envelope TCS (*rseABC*). It positively regulates genes *cpxP* and *degP* coding for the negative regulator (20). DegP has been shown to degrade misfolded periplasmic proteins, and its transcription is positively influenced by the stress σ-factor σ^24^ (35). We did not find any upregulation of *cpxRA*, but transcription of negative regulator gene *cpxP* (Δ*rfaD* mutant log_2_FC = 2.76; Δ*rfaG* mutant log_2_FC = 1.79) and periplasmic endoprotease gene *degP* (Δ*rfaD* mutant log_2_FC = 2.48) was enhanced.

Notably, we found significant upregulation of the well-known Rse/σ^24^ pathway (for *rpoE*, Δ*rfaD* mutant log_2_FC = 2.46 and Δ*rfaG* mutant log_2_FC = 1.87). The σ^24^ cell envelope stress response is primarily activated by misfolded outer membrane proteins in the periplasm (20). When these proteins are present, inner membrane-bound RseA is cleaved, resulting in release of σ^24^ into the cytosol. σ^24^ then induces expression of the downstream genes, including *rpoE*, *rseABC*, and *degP*.

The Rcs phosphorelay system is also known to respond to envelope stress (21, 36, 37). It was reported recently that RcsF monitors lateral interactions between LPS molecules. Upon disruption by cationic antimicrobial peptides such as polymyxin B resulting from loss of negatively charged phosphate groups on the LPS, the Rcs system activates the cell envelope stress response (30, 38). However, no Rcs-regulated genes were found to be differentially expressed in the transcriptome of the analyzed LPS mutants. Furthermore, polymyxin B had no effect on the upregulation of *rflP* (Fig. 6B).

### EDTA-mediated cell envelope stress is transmitted via σ^24^ and the Rcs phosphorelay to upregulate *rflP* expression.

The transcriptome analyses suggested that the Cpx cell envelope stress sensing system, as well as other factors or cell envelope stress sensing systems, may be involved in transcriptional activation of *rflP*. Thus, we next determined *rflP* expression levels upon addition of EDTA in various deletion mutants with respect to regulatory pathways putatively involved in *rflP* regulation. Growth of the TCS deletion mutants was not affected compared to that seen with the Wt (Fig. S5). Notably, *rflP* levels increased only slightly in the *rpoE* mutant under EDTA stress conditions, indicating that σ^24^ is involved in induction of *rflP* under cell envelope stress conditions. However, none of the tested regulatory pathways mutants displayed *rflP* expression levels comparable to those seen under the nonstimulated Wt conditions (Fig. 7D).

**FIG 8:**
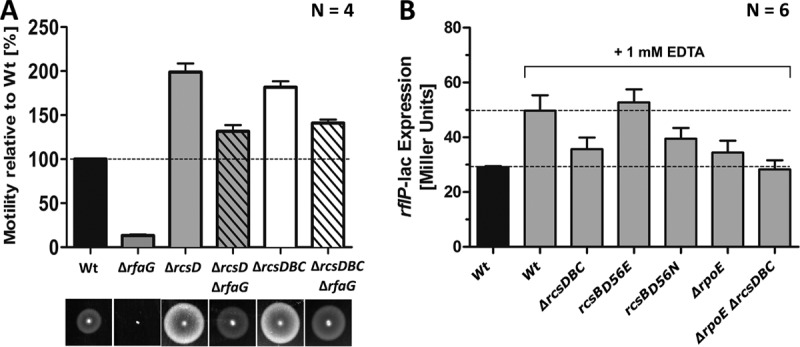
Influence of the Rcs pathway on the motility defect in the LPS mutants. (A) Swimming motility of the Wt strain, Δ*rfaG* mutants, and Δ*rfaG* Δ*rcs* double mutants assessed on semisolid agar after 4 h of incubation at 37°C. Bars represent means + standard errors of the means of results from 2 individual experiments (*n* = 4). (B) *rflP-lac* expression either in an RcsB mutant background that mimics phosphorylation (*rcsB*_*D56E*_*—rcsB*^on^) or in a mutant that is unable to be phosphorylated (*rcsB*_*D56N*_*—rcsB*^off^) upon addition of 1 mM EDTA. *Salmonella* Wt bacteria grown in LB served as controls. The bacteria were grown for 1.5 h in LB. EDTA (1 mM) was added, and bacteria were cultured for ~1.5 h prior to gene expression measurement. Bars represent means + standard errors of the means of results from 2 individual experiments (*n* = 6).

As stated above, the Rcs phosphorelay system has been shown to be involved in downregulation of motility upon disruption of the LPS in E. coli. Consistent with the previous findings in E. coli, the motility defect of a Δ*rfaG* mutant was rescued by additional deletion of *rcsD* or *rcsDBC* (Fig. 8A). To test whether the Rcs system is also involved in general upregulation of *rflP* expression under conditions of EDTA-mediated envelope stress, we determined *rflP* expression levels in a Δ*rcsDBC* mutant. Similarly to the phenotype of the *rpoE* mutant, *rflP* levels were enhanced only slightly under cell envelope stress conditions (Fig. 7D).

**FIG 9:**
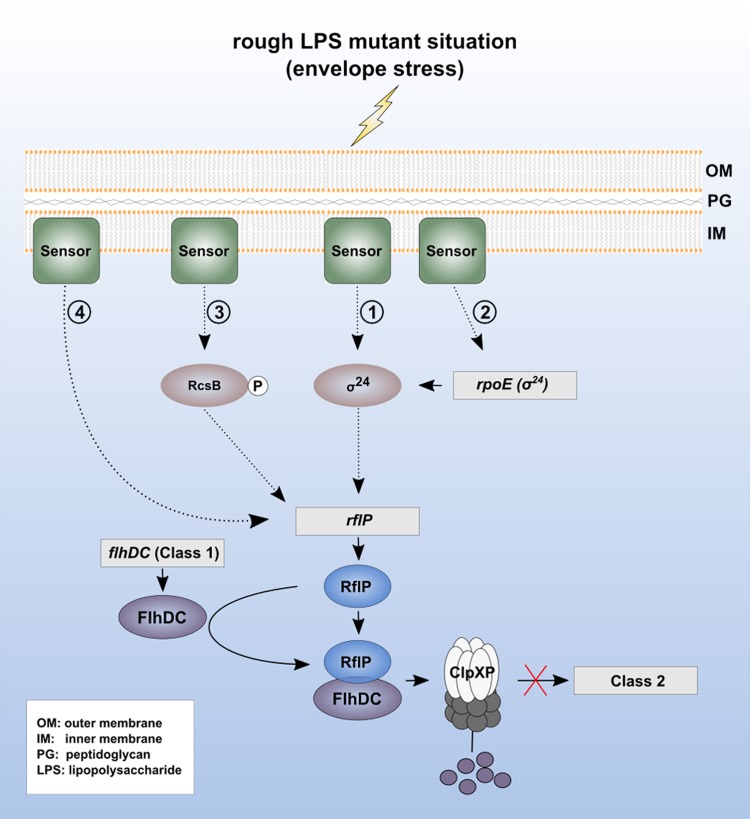
Model of envelope stress-mediated downregulation of flagellar gene expression. Cell envelope stress signals are sensed in the LPS mutant background by various sensory systems located in the membrane. The signal may be transmitted (i) directly via the alternative cell envelope stress sigma factor σ^24^, which is encoded by the *rpoE* gene, (ii) indirectly by enhancing transcription of *rpoE*, (iii) via the Rcs signaling pathway by phosphorylating RcsB, or (iv) via an unknown sensor and signaling pathway. RflP expression is then increased directly by σ^24^ via the activity of a σ^24^ binding sequence in the promoter region of *rflP*, by direct or indirect induction by RcsB-P, or by other factors. Increased levels of RflP protein led to degradation of the flagellar master regulator protein complex FlhD_4_C_2_ via ClpXP protease and subsequent downregulation of flagellar synthesis. Genes are indicated by gray boxes, and gene names are in italics.

The RcsB response regulator is phosphorylated upon activation of the Rcs phosphorelay system. We thus monitored *rflP* gene expression in a RcsB mutant that mimics phosphorylation (RcsB_D56E_) and in a mutant that prevents phosphorylation (RcsB_D56N_) (39). The RcsB_D56N_ (RcsB^OFF^) mutant failed to upregulate *rflP* expression under EDTA stress conditions compared to the Wt or the RcsB_D56E_ (RcsB^ON^) mutant (Fig. 8B). Notably, a double mutant with mutations of both the Rcs system and stress sigma factor σ^24^ (Δ*rcsDBC* Δ*rpoE*) did not activate *rflP* transcription under EDTA stress conditions (Fig. 8B). These results suggest that activation of both the Rcs phosphorelay system and the Rse/σ^24^ pathway is needed for full activation of *rflP* expression. We thus conclude that several redundant cell envelope sensing systems transmit and integrate signals corresponding to alterations of the cell envelope at the level of *rflP* expression, ultimately resulting in repression of flagellum production (Fig. 9).

## DISCUSSION

During host infection, *Salmonella* survives and replicates in vastly different anatomical compartments, such as in the gastrointestinal lumen or inside acidic host cell vacuoles, termed *Salmonella*-containing vacuoles (SCV). Accordingly, the bacteria employ a variety of sophisticated sensory systems and complex regulatory mechanisms to identify the spatiotemporal stage of infection and adapt to the various stressful environments. In particular, flagellum-mediated motility allows directed movement in the intestinal lumen but might be detrimental in later stages of infection or during intracellular survival. Biosynthesis of the large extracellular flagellar filament represents a serious metabolic burden and, in addition, constitutes a prime target for the immune system. Thus, bacteria tightly regulate flagellar biosynthesis in response to a plethora of environmental signals (5).

Here, we demonstrated that *Salmonella* senses various types of cell envelope stress, which subsequently trigger downregulation of motility and flagellar synthesis. Perturbations of the LPS structure exerted a strong inhibitory effect on the expression of flagellar genes from class 2 and class 3 promoters and a weak effect on the class 1 promoter that drives expression of the master regulatory operon, *flhDC*. Importantly, we found that flagellar gene expression was downregulated in the LPS truncation mutants primarily due to posttranscriptional regulation at the level of the FlhD_4_C_2_ complex.

Using an unbiased transposon mutagenesis approach, we identified the previously described anti-FlhD_4_C_2_ factor RflP as a key regulatory protein involved in downregulation of flagellar gene expression in the LPS truncation mutants. Importantly, we also found the ClpXP protease to be involved in this process. RflP-ClpXP-mediated regulation of FlhD_4_C_2_ is well established; RflP binds to the FlhD subunit and directs the master regulator FlhD_4_C_2_ with respect to ClpXP-dependent proteolytic degradation (12, 15). Thus, mutations in *rflP* or *clpXP* stabilize the FlhD_4_C_2_ protein complex and allow flagellar gene expression from class 2 promoters.

One of the major functions of the LPS layer is to control the stability of the outer membrane. Depletion of the LPS results in perturbations of the OM, which ultimately leads to leakage of periplasmic content into the extracellular space (22). Moreover, the composition of the OM is altered in LPS truncation mutants (22). In agreement with an earlier study (25), we argued that neither the altered LPS structure *per se* nor the mutated genes for LPS biosynthesis are directly responsible for the observed motility defect. Rather, we presumed that the alteration of LPS moieties caused general perturbations of the OM, which induced a general cell envelope stress response. For E. coli, it has been shown that the Rcs phosphorelay system, which senses outer membrane perturbations, is involved in the downregulation of flagella in LPS-defective mutants (25). Consistently, we found that the Rcs pathway upregulates *rflP* expression under EDTA envelope stress conditions and that this process depends on the phosphorylation state of the RcsB response regulator (Fig. 8B).

We further analyzed the induction of cell envelope sensory systems in more detail using transcriptome sequencing (RNA-seq)-based analysis and found significant upregulation of the Rse/σ^24^ pathway. The σ^24^ cell envelope stress response is primarily activated by the activity of misfolded outer membrane proteins in the periplasm (20). Activation of this pathway in the LPS mutants can be explained by the fact that the LPS subunits are synthesized but are not cross-linked and thus may accumulate in the periplasm. Interestingly, an *rpoE* deletion mutant was unable to upregulate *rflP* under conditions that perturbed the OM integrity using EDTA (Fig. 6). This result further confirmed the involvement of the σ^24^-dependent cell envelope stress response in downregulation of motility.

We also found that additional pathways besides flagellar motility and chemotaxis were downregulated in the LPS truncation mutants, in particular, pathways involving SPI-1- and SPI-2-associated genes, type I fimbriae, and the TCA cycle. In contrast, positively regulated genes were mapped to regulatory cascades and metabolic pathways. Interestingly, similar drastic changes in the gene expression patterns of flagella and chemotaxis genes were observed in E. coli when LPS synthesis was disrupted (40). This indicated that the existence of a nonmotile phenotype under envelope stress conditions is not restricted to *Salmonella* Typhimurium but might be a more general principle in gammaproteobacteria (40). Downregulation of the virulence-associated SPI-1 injectisome system might be connected to downregulation of the flagellar gene encoding FliZ. FliZ is needed for activation of the SPI-1-encoded injectisome in that it stabilizes the HilD SPI-1 master regulator (41).

As mentioned, *Salmonella* encounters various types of cell envelope stress during the course of infection. Upon *Salmonella* ingestion, antimicrobial peptides challenge the bacteria in the saliva. The bacteria travel through the digestive tract and encounter acidic pH in the stomach and bile salts before *Salmonella* reaches the intestine. In the intestine, antimicrobial peptides and the host microbiota and their products challenge the invading pathogen. Once *Salmonella* has breached the intestinal epithelial border, serum complement, as part of the immune system, attacks the bacteria. In the deeper organs, besides phagocytic cells, antimicrobial peptides and an acidic pH are found (42, 43). We thus tested flagellation and upregulation of *rflP* upon addition of EDTA, acidic pH, or serum complement as alternative means to induce cell envelope stress. EDTA is a chelator of positive metal ions such as Mg^2+^ and disrupts the OM (38), while pore formation mediated by the complement system might also induce the cell envelope stress response by perforation of the inner membrane (44). Expression of *rflP* was significantly upregulated by addition of EDTA and the complement but not by antimicrobials or detergents.

In summary, our results indicate that *Salmonella* responds to different types of envelope stress and might use these stresses as cues to determine the spatiotemporal stage of infection. At certain stages of the infection, it might be beneficial for *Salmonella* to downregulate flagellar synthesis in order to escape recognition by the immune system. In this respect, it appears possible that *rflP* is upregulated within the *Salmonella*-containing vacuole in response to cell envelope stress and limited nutrient levels. Consistently, upregulation of *rflP* under conditions that mimic *spi*-2 induction, as well as under starvation conditions, was reported before (13, 45). The various cell envelope stress signals would then result in activation of σ^24^ and the Rcs phosphorelay system, which in turn would increase expression of RflP. In support of that idea, a bioinformatics analysis of the *rflP* promoter region in E. coli suggested the presence of a putative σ^24^ binding sequence in the intergenic region between *nlpC* and *rflP* (46). After σ^24^-dependent activation of *rflP* gene expression, the RflP protein would subsequently target the flagellar master regulatory complex FlhD_4_C_2_ to ClpXP-mediated proteolysis and thereby downregulate flagellar synthesis (Fig. 8).

## MATERIALS AND METHODS

### Bacterial strains, plasmids, and media.

All bacterial strains used in this study are listed in Table S3 in the supplemental material. Cells were grown in lysogeny broth (LB) media (47) unless otherwise indicated. Cells harboring temperature-sensitive plasmid pKD46 for λ RED recombination were grown at 30°C, and the medium was supplemented with 0.2% arabinose and ampicillin. Gene deletions were constructed using λ RED recombination (48). The *rfaG* and *rfaD* mutations were introduced as described elsewhere (24). The generalized transducing phage of *S*. Typhimurium P22 *HT105*/*1 int-201* was used in all transductional crosses (49).

10.1128/mBio.00736-17.8 TABLE S3  Salmonella enterica serovar Typhimurium strains. Download TABLE S3, DOCX file, 0.1 MB. Copyright © 2018 Spöring et al. 2018 Spöring et al. This content is distributed under the terms of the Creative Commons Attribution 4.0 International license.

### Growth curve.

Bacteria were grown overnight at 37°C and adjusted to an optical density at 600 nm (OD_600_) of 0.001 in LB. A 200-μl volume was pipetted in a honeycomb multiwall plate. For measurement of growth, the honeycomb plate was incubated for the indicated duration, and the results were determined in a multiwell reader (Bioscreen). OD_600_ was measured every 15 min. The samples were blank corrected after measurement, and an unpaired *t* test was performed for statistical analysis using GraphPad Prism version 6.0 software.

### Motility and swarming assay.

Swimming motility was assessed on soft-agar plates (0.3% [wt/vol] agar) as described before (23). Briefly, bacteria had been streaked freshly on LB agar plates the day before and incubated overnight. Single colonies were stabbed into the agar, and the plates were incubated at 37°C for 4 to 5 h. Images were taken by scanning the soft-agar plates. The diameters of the swimming halos were analyzed by using NIH ImageJ 1.48v software and normalized to the Wt data. Swarming motility was assessed on swarm plates (0.6% [wt/vol] agar) as previously described (23). A 5-μl volume of the overnight culture was spotted on the swarm plate. The plates were incubated in a wet chamber for 5 to 6 h at 37°C. Images were taken by scanning the agar plates. The area of the swarming halos were analyzed by using NIH ImageJ 1.48v software and normalized to the Wt data. For statistical analysis, an unpaired *t* test was performed using GraphPad Prism version 6.0 software.

### SDS-PAGE and Western blotting.

Phase-locked FliC-ON bacterial cultures were grown until an OD_600_ of 0.9 to 1.2 was reached. Two milliliters of culture was used for harvesting cells (whole-cell lysate) and the supernatant by centrifugation. Proteins were precipitated by the use of 10% trichloroacetic acid, and the samples were resuspended in SDS sample buffer normalized to their OD_600_ (final concentration, 20 OD units/µl). Proteins were separated by SDS-PAGE, and FliC and DnaK were detected using anti-FliC and anti-DnaK antibodies, respectively, and ECL Plus Western blotting detection reagents (Amersham Biosciences, Inc.).

### Protein degradation assay.

Protein degradation was determined as described before with minor modifications (10). Bacteria harboring a chromosomal *flhC* 3× FLAG construct under the control of the native *flhDC* promoter were grown to mid-exponential phase in LB at 37°C. *De novo* protein synthesis was stopped by addition of spectinomycin (0.5 mg/ml) and chloramphenicol (0.012 mg/ml). Samples were taken at the indicated time points, and the bacteria were harvested by centrifugation at 4°C. The cell pellets were resuspended in 1 ml ice-cold water, and proteins were precipitated using 10% trichloroacetic acid and detected by Western blotting as described above.

### β-Galactosidase assay.

Analyses of gene expression levels were performed using transcriptional β-galactosidase fusions as described previously (9) with minor modifications. Bacterial cultures were grown in the presence or absence of EDTA until an OD_600_ of 0.9 to 1.2 was reached after 1.5 h of growth. For measuring the expression level in response to the complement, cells were mixed in a 1:1 ratio with human serum from volunteers and incubated for 30 min at 37°C. Heat-inactivated serum treated for 2 h at 56°C served as a control. Miller units were calculated according to the method previously reported by Miller (50). For statistical analysis, an unpaired *t* test was performed using GraphPad Prism version 6.0 software.

### RNA isolation and quantitative real-time PCR.

Gene expression analysis by quantitative real-time PCR was performed for every strain with at least four biological replicates and two technical replicates. Bacteria were grown until an OD_600_ of 0.9 to 1.2 with or without addition of EDTA was reached after 1.5 h of growth, and total RNA was isolated using an RNeasy kit (Qiagen). DNA was removed by DNase treatment using a Turbo DNA-free kit (Ambion). Reverse transcription and quantitative real-time PCR were performed using a SensiFast SYBR No-ROX One-Step kit (Bioline) in a Rotor-Gene Q LightCycler (Qiagen). Analysis of the relative mRNA level changes was done according to the method described by Pfaffl (51). MRNA levels were normalized to the mRNA levels of the *gmk*, *rpoD*, and *gyrB* reference genes as described before (52). For statistical analysis, an unpaired *t* test was performed using GraphPad Prism version 6.0 software.

### Scanning electron microscopy.

Bacteria were cultured overnight and then fixed in glutaraldehyde (2% final concentration) and stored at 4°C. After washing with TE buffer (20 mM Tris, 1 mM EDTA, pH 6.9) was performed, bacteria were placed onto poly-l-lysine-covered coverslips, dehydrated with a graded series of acetone concentrations, subjected to critical point drying with CO_2_, and sputter coated with gold palladium. Samples were imaged with a Zeiss Merlin field emission scanning electron microscope (FESEM) at an acceleration voltage of 5 kV using an Everhart-Thornley SE detector and an Inlens SE detector at a 25:75 ratio. Images were recorded with Zeiss SEM software version 5.05.

### NPN uptake assay.

Outer membrane distortion was analyzed using an NPN (1-N-phenylnaphtylamine; Sigma) uptake assay. The assay was conducted as described previously (29, 30). Briefly, bacterial cultures were grown until an OD_600_ of 0.5 was reached and were harvested by centrifugation. The cell pellet was washed in 5 mM HEPES (pH 7.2) and adjusted to an OD_600_ 0.5, and NPN was added to reach a final concentration of 10 µM. A 200-μl volume per sample was pipetted into a flat-bottom black 96-well plate (Greiner), and fluorescence (excitation wavelength [Ex], 350 nm; emission wavelength [Em], 420 nm) was measured for 10 min with 1-min intervals (Varioskan Flash Microplate Reader, Thermo Fisher). Two technical replicates were measured per biological sample. End point measurement after 10 min was blank corrected and normalized to the Wt data. For statistical analysis, an unpaired *t* test was performed using GraphPad Prism version 6.0 software.

### Tn*5* random transposon mutagenesis.

Random transposon mutagenesis was done using an EZ-Tn5 <R6Kγori/KAN-2>Tnp transposome kit (Epicentre) using the manual instructions with minor modifications and recipient strain EM3731 harboring a *fliL*::*lac* fusion and lacking *rfaG*. Electrocompetent cells were prepared by growing the cells to an OD_600_ of 0.9 and washing twice with ice-cold MilliQ H_2_O. Cells were transformed with the EZ-Tn5 transposome and recovered in LB medium. Transformants were plated on lactose fermenter indicator agar plates (triphenyl tetrazolium chloride [TTC] agar) supplemented with kanamycin and were incubated overnight at 37°C. Lac-positive colonies were identified, and the transposon insertion site was determined using DNA sequencing analyses.

### Transcriptome analysis.

For transcriptome analysis, mRNA of two independent cultures per strain grown to an OD_600_ of 1.3 was isolated using an RNeasy kit (Qiagen) as described in the instructions. The quality was assessed using an Agilent RNA 6000 Nano kit and an Agilent 2100 Bioanalyzer (Agilent Technologies). CDNA preparation and deep sequencing were performed as described previously (53). Sequence reads were separated according to their barcodes and mapped to the genome sequence of the reference genome Salmonella enterica subsp. enterica serovar Typhimurium LT2 (GenBank accession number AE006468.2) using Stampy (54). For differential gene expression analysis, the R package DESeq (55) was employed. Differentially expressed genes were identified using the *nbinomTest* function based on the negative binomial model. Genes were considered to have been differentially expressed if they matched the following criteria: (i) upregulation or downregulation by a factor of 2 (log_2_FC: −1/1) and (ii) adjusted *P* value of >13.

### Accession number(s).

All raw and processed expression data were submitted to GEO under GenBank accession number GSE87447 (https://www.ncbi.nlm.nih.gov/geo/query/acc.cgi?acc=GSE87447).

10.1128/mBio.00736-17.9 TABLE S4  Oligonucleotides used in this study. Download TABLE S4, DOCX file, 0.1 MB. Copyright © 2018 Spöring et al. 2018 Spöring et al. This content is distributed under the terms of the Creative Commons Attribution 4.0 International license.
