# Supplementary material for: Regulation of Flagellum Biosynthesis in Response to Cell Envelope Stress in Salmonella enterica Serovar Typhimurium
Source: mBio. 2018 May 1;9(3):e00736-17. doi: 10.1128/mBio.00736-17 (PMC5930307; doi:10.1128/mBio.00736-17)
Supplement: FIG S1 [file mbo002183865sf1.pdf]

Figure S1

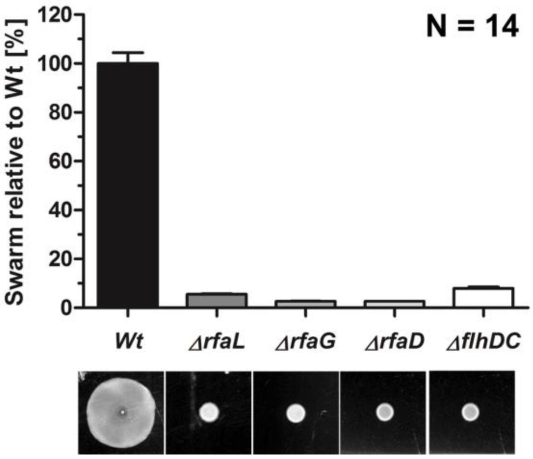

Fig. S1: Swarming motility of the *Salmonella* LPS mutants. Swarming motility was analyzed on plates containing 0.6% agar after 8 h incubation at 37 °C. Bars represent mean + SEM of 2 individual experiments (n=14).
