# Supplementary material for: Regulation of Flagellum Biosynthesis in Response to Cell Envelope Stress in Salmonella enterica Serovar Typhimurium
Source: mBio. 2018 May 1;9(3):e00736-17. doi: 10.1128/mBio.00736-17 (PMC5930307; doi:10.1128/mBio.00736-17)
Supplement: FIG S2 [file mbo002183865sf2.pdf]

Figure S2

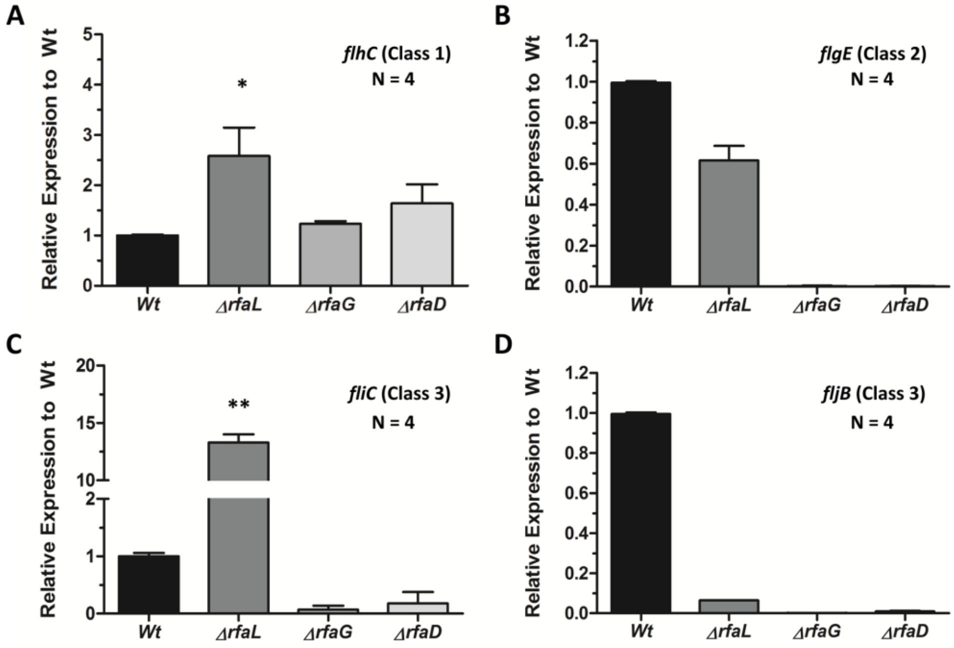

Fig. S2: Relative gene expression of the flagellar genes in *Salmonella* LPS mutants. Relative *flhC* (Class 1), *flgE* (Class 2) and *fliC* (Class 3) gene expression levels of the LPS mutant strains Δ*rfaL*, Δ*rfaG* and Δ*rfaD* compared to Wt *Salmonella* analyzed by qRT-PCR. Bars represent mean + SEM of 2 individual experiments (n=4). * P ≤ 0.05 ** P ≤ 0.01.
