# Supplementary material for: Regulation of Flagellum Biosynthesis in Response to Cell Envelope Stress in Salmonella enterica Serovar Typhimurium
Source: mBio. 2018 May 1;9(3):e00736-17. doi: 10.1128/mBio.00736-17 (PMC5930307; doi:10.1128/mBio.00736-17)
Supplement: TABLE S1 [file mbo002183865st1.docx]

**Table S1: Chromosomal EZ-*Tn*5 transposon integration sites**

| **Clone** | **Gene** | **Position 5'ATG** | **Description** |
| --- | --- | --- | --- |
| G3, G6 | *clpX* | (+ 585 bp), (+49 bp) | ATP-binding subunit ClpX of the ATP-dependent protease ClpXP |
| G4, G13 | *atpD* | (+1355 bp), (+42 bp) | F-Type H+ transporting subunit beta |
| G7 | *nlpC* | (+59 bp) | Lipoprotein, unknown function |
| G9 | *nudE* | (+173 bp) | ADP-ribose diphosphatase |
| G12 | *rflP* | (-93 bp) | anti-FlhD_4_C_2_ factor |
