# Supplementary material for: Regulation of Flagellum Biosynthesis in Response to Cell Envelope Stress in Salmonella enterica Serovar Typhimurium
Source: mBio. 2018 May 1;9(3):e00736-17. doi: 10.1128/mBio.00736-17 (PMC5930307; doi:10.1128/mBio.00736-17)
Supplement: FIG S3 [file mbo002183865sf3.pdf]

Figure S3

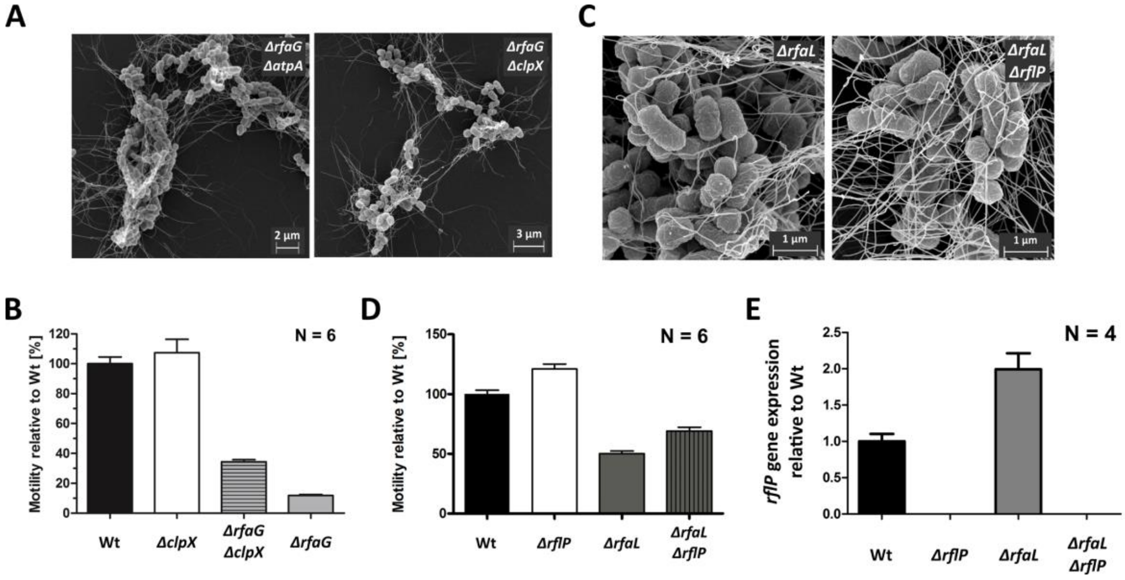

Fig. S3: Deletion of *clpX* and *atpA* partially restores motility and flagellation of LPS mutants and deletion of *rflP* modulates flagellation of *ΔrfaL* mutant without affecting motility. A) Scanning electron microscopy of the LPS mutant strains Δ*rfaG* in absence of *atpA* (left) and *clpX* (right). B) Swimming motility of Wt, a Δ*clpX* mutant and LPS mutant Δ*rfaG* in absence and presence of *clpX* assessed on semi-solid agar after 4 h incubation at 37 °C. Bars represent mean + standard error of the mean (SEM) of 2 individual experiments (n=6). C) Scanning electron microscopy of the *ΔrfaL* and *ΔrfaL ΔrflP* mutant. D) Swimming motility of the Wt and *ΔrfaL* mutant strain in absence and presence of *rflP* assessed on semi-solid agar after 4 h incubation at 37 °C. Bars represent mean + SEM of 2 individual experiments (n=6). E) Relative *rflP* gene expression levels of the LPS mutant strains *ΔrfaL* and *ΔrfaL ΔrflP* compared to Wt *Salmonella* analyzed by qRT-PCR. Bars represent mean + SEM of 2 individual experiments (n=4).
