# Supplementary material for: Regulation of Flagellum Biosynthesis in Response to Cell Envelope Stress in Salmonella enterica Serovar Typhimurium
Source: mBio. 2018 May 1;9(3):e00736-17. doi: 10.1128/mBio.00736-17 (PMC5930307; doi:10.1128/mBio.00736-17)
Supplement: FIG S4 [file mbo002183865sf4.pdf]

Figure S4

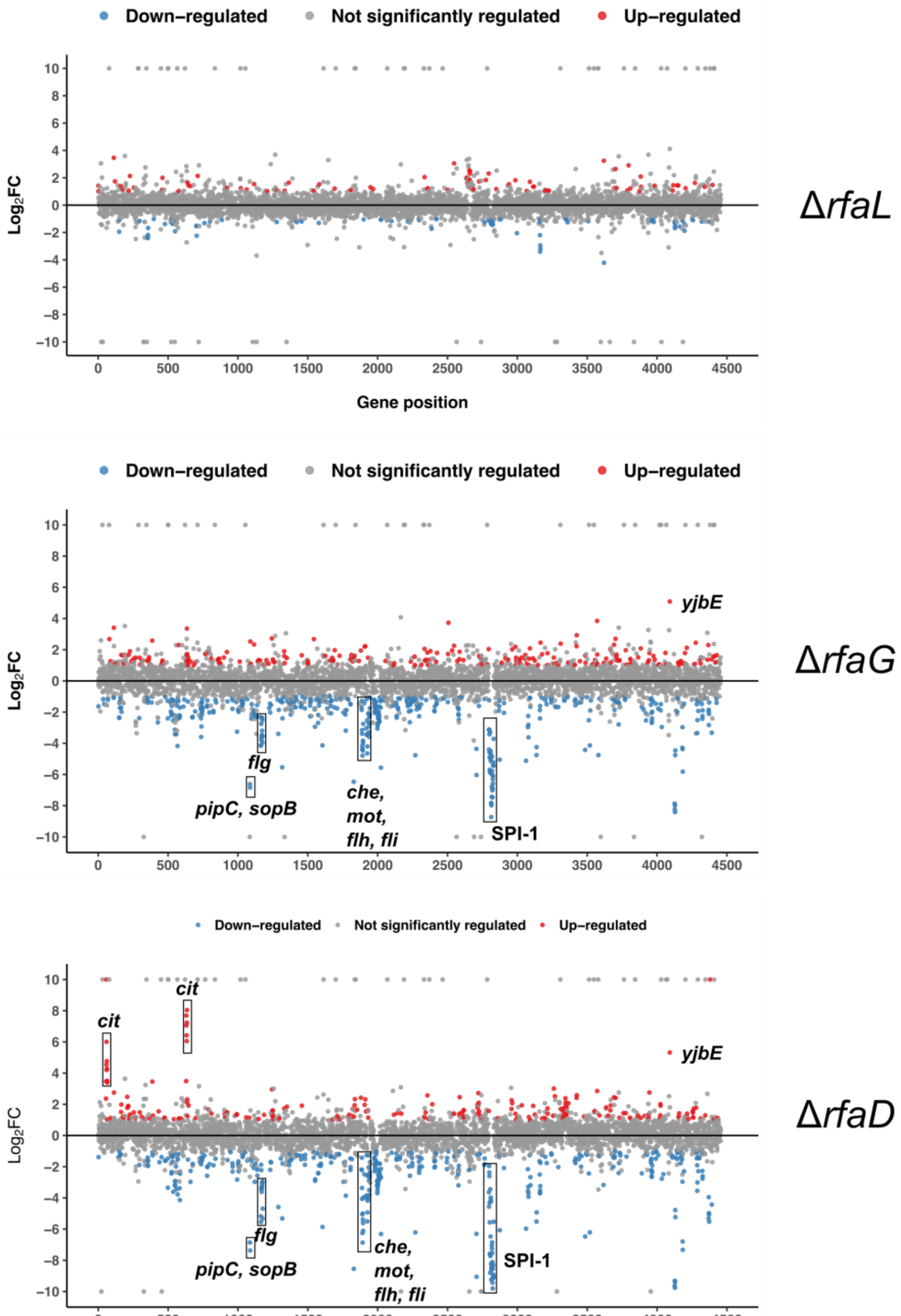

Fig. S4: Scatter plot of the *ΔrfaL*, *ΔrfaG* and *ΔrfaD* transcriptomes. Significantly and differentially regulated genes with a Log_2FC of ≤ 1 ≥ are shown in red (upregulated) and blue (downregulated). Not significantly and differentially regulated genes are shown in grey. Prominent differentially regulated genes and gene clusters are indicated by boxes.
