# Supplementary material for: Regulation of Flagellum Biosynthesis in Response to Cell Envelope Stress in Salmonella enterica Serovar Typhimurium
Source: mBio. 2018 May 1;9(3):e00736-17. doi: 10.1128/mBio.00736-17 (PMC5930307; doi:10.1128/mBio.00736-17)
Supplement: FIG S5 [file mbo002183865sf5.pdf]

Figure S5

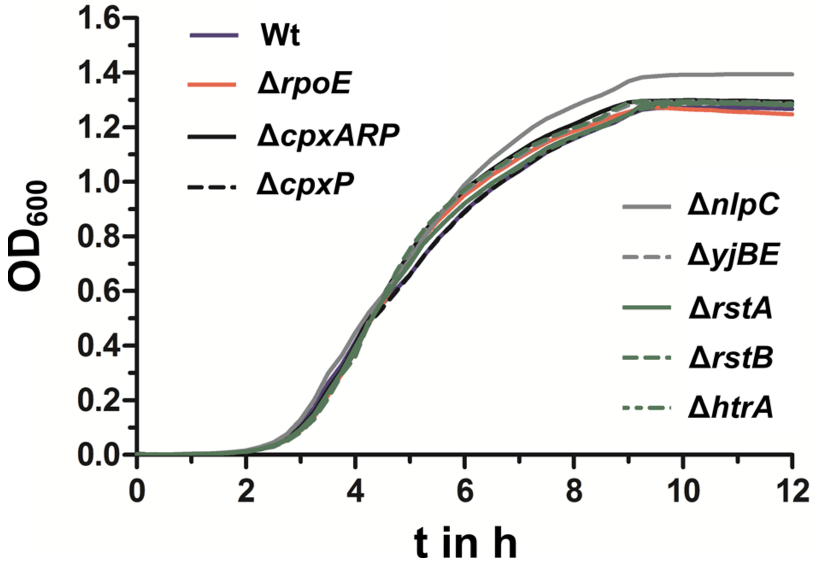

Fig. S5: Growth curves of putative sensor mutants. Growth was monitored over time at 37 °C of the deletion mutants *cpxAR cpxP* (Δ*cpxARP*), *cpxP, nlpC, yjbE, rstA, rstB, htrA* compared to the Wt. Curves represent mean of 1 individual experiments (n=8).
