# Supplementary material for: Regulation of Flagellum Biosynthesis in Response to Cell Envelope Stress in Salmonella enterica Serovar Typhimurium
Source: mBio. 2018 May 1;9(3):e00736-17. doi: 10.1128/mBio.00736-17 (PMC5930307; doi:10.1128/mBio.00736-17)
Supplement: TABLE S2 [file mbo002183865st2.docx]

**Table S2: *In vitro* transcriptome analysis of the significantly up- and downregulated genes in the LPS mutants compared to the Wt**. Genes were considered being significantly differentially regulated when matching the criteria: (i) adjusted *P* value >13; (ii) Log_2_FC >1 or < -1 respectively.

| **Gene** | **Name** | | **Description** | **Log_2_FC** | | |
| --- | --- | --- | --- | --- | --- | --- |
| **Downregulated** | | |  | **Δ*rfaL*** | **Δ*rfaG*** | **Δ*rfaD*** |
| STM0002 | | *thrA* | bifunctional aspartokinase / homoserine dehydrogenase 1 |  | -1.63 | -1.39 |
| STM0035 | | *-* | arylsulfatase |  | -1.17 |  |
| STM0042 | | *-* | sodium galactoside symporter |  | -1.48 |  |
| STM0070 | | *caiD* | carnitinyl-CoA dehydratase |  | -1.21 |  |
| STM0113 | | *leuA* | 2-isopropylmalate synthase |  | -1.08 |  |
| STM0152 | | *aceE* | pyruvate dehydrogenase | -1.94 | -2.35 | -1.02 |
| STM0153 | | *aceF* | pyruvate dehydrogenase dihydrolipoyltransacetylase subunit E2 | -1.09 | -2.37 | -1.35 |
| STM0154 | | *lpdA* | pyruvate dehydrogenase lipoamide dehydrogenase subunit E3 |  | -1.41 |  |
| STM0158 | | *acnB* | bifunctional aconitate hydratase 2/2-methylisocitrate dehydratase |  | -1.73 |  |
| STM0161 | | *kdgT* | 2-keto-3-deoxygluconate permease |  | -1.05 | -1.24 |
| STM0162 | | *-* | inner membrane protein |  | -1.22 | -1.19 |
| STM0166 | | *speE* | spermidine synthase |  | -1.73 |  |
| STM0169 | | *gcd* | glucose dehydrogenase |  | -1.99 |  |
| STM0304 | | *sinR* | HTH-type transcriptional regulator |  | -2.39 | -1.88 |
| STM0306 | | *-* | adhesin/invasin protein PagN | -1.16 |  |  |
| STM0366 | | *yahO* | periplasmic protein |  | -1.08 | -1.58 |
| STM0368 | | *prpB* | 2-methylisocitrate lyase | -2.34 | -1.22 | -1.41 |
| STM0369 | | *prpC* | 2-methylcitrate synthase | -2.41 | -1.39 | -1.95 |
| STM0370 | | *prpD* | 2-methylcitrate dehydratase | -2.18 |  | -1.92 |
| STM0371 | | *prpE* | propionate--CoA ligase | -1.67 |  | -1.63 |
| STM0396 | | *sbcD* | ATP-dependent dsDNA exonuclease |  | -1.39 |  |
| STM0402 | | *-* | thiol-alkyl hydroperoxide reductase |  | -1.01 | -1.08 |
| STM0403 | |  | acyl carrier protein phosphodiesterase |  |  | -1.27 |
| STM0413 | | *tsx* | nucleoside-specific channel-forming protein | -1.01 |  |  |
| STM0442 | | *cyoB* | cytochrome o ubiquinol oxidase subunit I |  | -2.66 |  |
| STM0453 | | *ybaV* | DNA uptake protein (competence protein ComEA) |  |  | -1.71 |
| STM0465 | | *ybaY* | outer membrane lipoprotein |  |  | -1.38 |
| STM04870 | | *-* | hypothetical protein | -1.38 |  |  |
| STM0487.S | | *htpG* | chaperone |  | -1.03 | -1.63 |
| **Gene** | **Name** | | **Description** | **Log_2_FC** | | |
| **Downregulated** | | |  | **Δ*rfaL*** | **Δ*rfaG*** | **Δ*rfaD*** |
| STM04875 | |  | hypothetical protein |  |  | -1.60 |
| STM0501 | | *ybbK* | inner membrane protein |  | -1.28 | -1.49 |
| STM05010 | | *-* | hypothetical protein |  | -2.40 | -3.35 |
| STM0517 | | *gcl* | glyoxylate carboligase |  | -1.41 |  |
| STM0518 | | *gip* | hydroxypyruvate isomerase |  | -1.97 |  |
| STM0522 | | *allP* | allantoin permease |  | -2.22 | -2.44 |
| STM0523 | | *allB* | allantoinase | -1.01 | -2.07 | -2.33 |
| STM0524 | | *ybbY* | uraxil/xanthine transporter |  | -1.61 |  |
| STM0526 | | *ylbA* | hypothetical protein ((S)-ureidoglycine aminohydrolase) |  | -1.48 | -1.29 |
| STM0527 | | *allC* | allantoate amidohydrolase | -1.30 | -1.26 | -1.38 |
| STM0528 | | *allD* | ureidoglycolate dehydrogenase | -1.36 | -1.20 | -1.69 |
| STM05320 | | *-* | hypothetical protein |  | -2.55 | -3.61 |
| STM0543 | | *fimA* | type-1 fimbrial protein subunit A |  | -3.13 | -3.22 |
| STM0544 | | *fimI* | fimbrin-like protein | -1.91 | -3.42 | -3.15 |
| STM0545 | | *fimC* | chaperone protein |  | -2.21 | -2.91 |
| STM0546 | | *fimD* | outer membrane usher protein |  | -1.29 | -1.24 |
| STM0549 | | *fimZ* | transcriptional regulator | -1.15 | -2.37 | -1.99 |
| STM0552 | | *fimW* | fimbrial regulatory protein |  | -3.42 | -3.45 |
| STM0557 | | *-* | inner membrane protein |  | -1.58 |  |
| STM05625 | | *-* | hypothetical protein |  | -4.18 | -3.78 |
| STM0564 | | *-* | oxidoreductase |  | -1.06 | -1.52 |
| STM0567 | | *-* | DNA repair ATPase |  | -1.30 | -1.19 |
| STM0573 | | *-* | inner membrane protein |  | -2.38 | -3.38 |
| STM0574 | | *-* | PTS system mannose-specific transporter subunit IID |  | -2.27 | -2.37 |
| STM0575 | | *-* | PTS system mannose-specific transporter subunit IIC |  | -2.22 | -4.14 |
| STM0576 | | *-* | PTS system mannose-specific transporter subunit IIB |  | -1.71 | -2.45 |
| STM0600 | | *cstA* | carbon starvation protein |  | -1.65 |  |
| STM0605 | | *ybdN* | 3'-phosphoadenosine 5'-phosphosulfate sulfotransferase |  | -1.19 |  |
| STM0631 | | *ybeM* | hydrolase |  | -1.32 | -1.54 |
| STM0649.S | | *-* | putative hydrolase N-terminus |  |  | -2.97 |
| STM0650 | | *-* | putative hydrolase C-terminus |  |  | -2.27 |
| STM0663 | | *gltK* | glutamate/aspartate ABC transporter permease |  | -2.34 |  |
| STM0665 | | *gltI* | glutamate/aspartate ABC transporter substrate-binding protein |  | -2.30 | -1.82 |
| STM0690 | | *citB* | citrate utilization protein B |  | -1.96 |  |
| STM0699 | | *-* | cytoplasmic protein | -1.54 |  |  |
| **Gene** | **Name** | | **Description** | **Log_2_FC** | | |
| **Downregulated** | | |  | **Δ*rfaL*** | **Δ*rfaG*** | **Δ*rfaD*** |
| STM0700 | | *potE* | APC family putrescine/ornithine antiporter | -1.50 |  |  |
| STM0701 | | *speF* | ornithine decarboxylase | -2.24 |  |  |
| STM0730 | | *gltA* | citrate synthase |  | -1.80 | -1.69 |
| STM0732 | | *sdhC* | succinate dehydrogenase cytochrome b556 subunit |  | -2.40 | -1.85 |
| STM0733 | | *sdhD* | succinate dehydrogenase cytochrome b556 small membrane subunit |  | -3.59 | -1.41 |
| STM0734 | | *sdhA* | succinate dehydrogenase flavoprotein subunit |  | -1.37 |  |
| STM0735 | | *sdhB* | succinate dehydrogenase iron-sulfur subunit |  | -2.92 |  |
| STM0736 | | *sucA* | 2-oxoglutarate dehydrogenase decarboxylase subunit E1 |  | -1.78 |  |
| STM0737 | | *sucB* | 2-oxoglutarate dehydrogenase dihydrolipoyltranssuccinase subunit E2 |  | -1.68 |  |
| STM0738 | | *sucC* | succinyl-CoA ligase subunit beta |  | -1.95 | -1.08 |
| STM0739 | | *sucD* | succinyl-CoA ligase subunit alpha |  | -1.41 |  |
| STM0740 | | *cydA* | cytochrome d terminal oxidase polypeptide subunit I |  | -2.14 | -1.57 |
| STM0741 | | *cydB* | cytochrome d terminal oxidase polypeptide subunit II |  | -1.87 | -1.44 |
| STM0742 | | *ybgT* | outer membrane lipoprotein |  | -1.14 |  |
| STM0743 | | *ybgE* | inner membrane protein |  | -1.72 |  |
| STM0761 | | *-* | fumarate hydratase subunit beta |  |  | -1.29 |
| STM0762 | | *-* | fumarate hydratase subunit alpha | -1.31 |  |  |
| STM0781 | | *modA* | molybdate ABC transporter substrate-binding protein ModA |  | -1.66 | -2.58 |
| STM0782 | | *modB* | molybdate ABC transporter permease ModB |  | -1.57 | -2.00 |
| STM0783 | | *modC* | molybdate ABC transporter ATP-binding protein ModC |  | -1.25 | -2.03 |
| STM0804 | | *moaC* | cyclic pyranopterin monophosphate synthase accessory protein | -1.19 |  |  |
| STM0800 | | *slrP* | E3 ubiquitin-protein ligase SlrP |  | -2.02 | -1.83 |
| STM0829 | | *glnP* | glutamine ABC transporter permease |  | -1.92 |  |
| STM0830 | | *glnH* | glutamine ABC transporter substrate-binding protein |  | -2.02 | -1.91 |
| STM0844 | | *pflE* | pyruvate formate lyase activating enzyme |  | -1.21 | -1.49 |
| STM0849 | | *yliB* | glutathione ABC transporter substrate-binding protein (GsiB) |  | -1.38 |  |
| STM0891 | | *artP* | arginine ABC transporter ATP-binding protein ArtP | -1.08 |  |  |
| STM0928 | | *nanH* | sialidase |  | -1.31 | -1.57 |
| STM0951 | | *-* | cytoplasmic protein |  |  | -1.07 |
| STM0964 | | *dmsA* | anaerobic dimethyl sulfoxide reductase subunit A |  | -1.10 | -1.78 |
| STM0965 | | *dmsB* | anaerobic dimethyl sulfoxide reductase subunit B |  | -1.79 | -2.31 |
| **Gene** | **Name** | | **Description** | **Log_2_FC** | | |
| **Downregulated** | | |  | **Δ*rfaL*** | **Δ*rfaG*** | **Δ*rfaD*** |
| STM0966 | | *dmsC* | anaerobic dimethyl sulfoxide reductase subunit C |  | -1.24 | -1.99 |
| STM0994 | | *mukB* | chromosome partition protein |  | -1.36 |  |
| STM0999 | | *ompF* | outer membrane protein F |  | -3.26 | -2.83 |
| STM1008.S | | *-* | hypothetical protein |  | -1.88 | -1.66 |
| STM1009 | | *-* | exodeoxyribonuclease |  | -1.86 | -1.85 |
| STM1010 | | *-* | hypothetical protein |  | -1.98 | -2.30 |
| STM1010.1n | | *-* | hypothetical protein |  |  | -2.25 |
| STM1011 | | *-* | hypothetical protein |  |  | -2.00 |
| STM1032 | | *-* | hypothetical protein |  | -1.22 |  |
| STM1033 | | *-* | Clp protease-like protein |  | -1.62 | -1.16 |
| STM1034 | | *-* | recombinase A |  | -1.39 |  |
| STM1035 | | *-* | ATP-binding sugar transporter-like protein |  | -1.33 |  |
| STM1036 | | *-* | minor tail protein |  | -1.50 | -1.38 |
| STM1037 | | *-* | minor tail protein |  | -1.47 |  |
| STM1038 | | *-* | major tail protein |  | -1.31 | -1.30 |
| STM1039 | | *-* | minor tail protein |  | -1.86 |  |
| STM1049 | | *-* | tail fiber protein |  | -1.06 |  |
| STM1055 | | *-* | hypothetical protein |  |  | -1.09 |
| STM1070 | | *ompA* | outer membrane protein A |  | -1.17 | -1.60 |
| STM1090 | | *pipC* | chaperone protein SigE |  | -6.62 | -6.86 |
| STM1091 | | *sopB* | inositol phosphate phosphatase (vT3SS effector) |  | -6.84 | -7.37 |
| STM1101 | | *hpaG* | 4-hydroxyphenylacetate catabolism protein |  | -2.43 | -1.61 |
| STM1102 | | *hpaE* | 5-carboxymethyl-2-hydroxymuconate semialdehyde dehydrogenase |  | -2.19 | -1.45 |
| STM1103 | | *hpaD* | 3,4-dihydroxyphenylacetate 2,3-dioxygenase |  | -1.49 |  |
| STM1117 | | *agp* | glucose-1-phosphatase/inositol phosphatase |  | -2.05 | -2.08 |
| STM1123 | | *-* | periplasmic protein |  | -1.90 |  |
| STM1126 | | *-* | riboflavin biosynthesis protein |  | -2.13 | -2.97 |
| STM1131 | | *-* | outer membrane protein |  | -2.28 | -1.81 |
| STM1145 | | *csgC* | curli assembly protein |  | -1.55 | -2.19 |
| STM1146 | | *ymdA* | hypothetical protein |  | -2.29 | -2.57 |
| STM1171 | | *flgN* | FlgK/FlgL export chaperone (flagellum) |  | -4.13 | -5.18 |
| STM1172 | | *flgM* | anti-sigma-28 factor |  | -4.15 | -5.54 |
| STM1173 | | *flgA* | flagellar basal body P-ring formation protein FlgA |  | -2.33 | -1.87 |
| STM1174 | | *flgB* | flagellar basal body rod protein |  | -2.76 | -2.22 |
| STM1175 | | *flgC* | flagellar basal body rod protein |  | -3.80 | -3.42 |
| STM1176 | | *flgD* | flagellar basal body rod modification protein |  | -3.63 | -3.38 |
| STM1177 | | *flgE* | flagellar hook protein |  | -3.60 | -3.61 |
| **Gene** | **Name** | | **Description** | **Log_2_FC** | | |
| **Downregulated** | | |  | **Δ*rfaL*** | **Δ*rfaG*** | **Δ*rfaD*** |
| STM1178 | | *flgF* | flagellar basal body rod protein |  | -3.53 | -3.23 |
| STM1179 | | *flgG* | flagellar basal body rod protein |  | -3.47 | -3.34 |
| STM1180 | | *flgH* | flagellar basal body L-ring protein |  | -3.19 | -3.05 |
| STM1181 | | *flgI* | flagellar basal body P-ring protein |  | -2.37 | -3.02 |
| STM1182 | | *flgJ* | peptidoglycan hydrolase |  | -2.52 | -2.94 |
| STM1183 | | *flgK* | flagellar hook-associated protein |  | -3.95 | -5.32 |
| STM1184 | | *flgL* | flagellar hook-associated protein |  | -3.54 | -4.69 |
| STM1232 | | *purB* | adenylosuccinate lyase |  | -1.74 |  |
| STM1238 | | *icdA* | isocitrate dehydrogenase |  | -1.46 |  |
| STM1239 | | *-* | cytoplasmic protein |  | -2.03 | -2.20 |
| STM1242 | | *envE* | lipoprotein |  |  | -1.70 |
| STM1246 | | *pagC* | virulence membrane protein |  | -1.55 | -1.72 |
| STM1289 | | *yeaD* | aldose 1-epimerase-like protein | -1.23 |  |  |
| STM1296 | | *ydjA* | oxidoreductase |  | -1.20 |  |
| STM1300 | | *-* | periplasmic protein |  | -3.40 | -4.59 |
| STM1304 | | *astA* | arginine succinyltransferase |  | -1.86 |  |
| STM1316 | | *celF* | phospho-beta-glucosidase |  | -1.31 |  |
| STM1318 | | *katE* | catalase |  | -1.27 | -1.48 |
| STM1328 | | *-* | outer membrane protein |  | -5.54 | -5.32 |
| STM1329 | | *-* | inner membrane protein |  | -1.37 |  |
| STM1330 | | *-* | DNA/RNA non-specific endonuclease |  | -2.40 | -1.79 |
| STM1333 | | *thrS* | threonine--tRNA ligase |  | -1.05 |  |
| STM1349 | | *pps* | phosphoenolpyruvate synthase |  | -1.03 |  |
| STM1366 | | *-* | hypothetical protein |  | -1.30 |  |
| STM1379 | | *orf48* | amino acid permease |  | -1.39 | -1.90 |
| STM1381 | | *orf245* | cytoplasmic protein |  | -1.18 | -1.66 |
| STM1383 | | *ttrA* | tetrathionate reductase subunit A |  |  | -1.20 |
| STM1391 | | *ssrB* | secretion system transcriptonal activator SsrB (vT3SS) |  | -2.15 | -1.57 |
| STM1392 | | *ssrA* | secretion system sensor kinase SsrA (vT3SS) |  | -1.87 | -1.16 |
| STM1397 | | *sseA* | secretion system chaperone (vT3SS) |  | -1.50 | -1.52 |
| STM1398 | | *sseB* | secreted effector protein (vT3SS) |  | -1.39 |  |
| STM1400 | | *sseC* | secreted effector protein (vT3SS) |  | -1.16 | -1.05 |
| STM1415 | | *ssaN* | secretion system apparatus ATP synthase (vT3SS) |  | -1.34 |  |
| STM1428 | | *ydhC* | MFS family transport protein |  | -1.70 |  |
| STM1431 | | *sodB* | superoxide dismutase |  | -1.02 |  |
| STM1436 | | *nemA* | N-ethylmaleimide reductase | -1.25 | -1.24 | -1.26 |
| STM1451 | | *gst* | glutathionine S-transferase |  |  | -1.28 |
| STM1468 | | *fumA* | class I fumarate hydratase |  | -1.66 |  |
| **Gene** | **Name** | | **Description** | **Log_2_FC** | | |
| **Downregulated** | | |  | **Δ*rfaL*** | **Δ*rfaG*** | **Δ*rfaD*** |
| STM1469 | | *fumC* | class II fumarate hydratase |  | -1.65 | -1.53 |
| STM1473 | | *ompN* | outer membrane protein N | -1.20 | -1.45 | -1.31 |
| STM1480 | | *pntB* | NAD(P) transhydrogenase subunit beta |  | -1.02 |  |
| STM1497 | | *-* | dimethyl sulfoxide reductase subunit B |  | -1.76 | -1.88 |
| STM1498 | | *-* | dimethyl sulfoxide reductase subunit A |  | -1.08 | -1.49 |
| STM1499 | |  | dimethyl sulfoxide reductase subunit A |  |  | -1.24 |
| STM1537 | | *-* | Ni/Fe hydrogenase 1 b-type cytochrome subunit |  | -1.58 |  |
| STM1538 | | *-* | hydrogenase-1 large subunit |  | -2.26 |  |
| STM1539 | | *-* | hydrogenase-1 small subunit |  | -2.27 |  |
| STM1542 | | *-* | zinc-binding dehydrogenase |  | -1.16 |  |
| STM1543 | | *-* | transporter |  | -1.32 |  |
| STM1562 | | *hdeB* | acid stress chaperone |  |  | -1.42 |
| STM1567 | | *adhP* | alcohol dehydrogenase |  |  | -1.07 |
| STM1569 | | *fdnH* | formate dehydrogenase-N, Fe-S subunit beta |  | -1.83 |  |
| STM1570 | | *fdnG* | molybdopterin oxidoreductases |  | -1.77 |  |
| STM1572 | | *nmpC* | outer membrane porin protein (OmpD) |  | -2.94 | -2.91 |
| STM1573.Sc | | *-* | hypothetical protein |  |  | -2.81 |
| STM1578 | | *narY* | nitrate reductase 2 subunit beta |  | -1.21 |  |
| STM1593 | | *srfA* | SsrAB activated protein |  | -1.12 | -1.15 |
| STM1594 | | *srfB* | SsrAB activated protein |  | -2.69 | -2.43 |
| STM1595 | | *srfC* | SsrAB activated protein |  | -2.19 | -2.40 |
| STM1598 | | *ydcR* | GntR family regulatory protein |  | -1.32 |  |
| STM1599 | | *pdgL* | periplasmic dipeptidase | -1.31 |  |  |
| STM1601 | | *ugtL* | membrane protein |  | -1.58 | -1.93 |
| STM1612 | | *-* | aminopeptidase SgcX |  |  | -1.37 |
| STM1613 | | *-* | PTS system transporter subunit IIB |  | -1.24 | -1.86 |
| STM1614 | | *-* | PTS system transporter subunit IIC |  |  | -1.94 |
| STM1615 | | *-* | nucleoside triphosphatase |  | -1.32 | -1.80 |
| STM1626 | | *trg* | methyl-accepting chemotaxis protein III |  | -4.13 | -5.86 |
| STM1627 | | *-* | alcohol dehydrogenase class III |  | -1.30 |  |
| STM1640 | | *ydcF* | inner membrane protein |  | -1.01 |  |
| STM1652 | | *ynaF* | universal stress protein F |  |  | -1.26 |
| STM1654 | | *ydaO* | tRNA 2-thiocytidine biosynthesis protein (TtcA) |  |  | -1.15 |
| STM1657 | | *-* | methyl-accepting chemotaxis protein |  | -1.36 | -1.17 |
| STM1662 | | *ynaJ* | inner membrane protein |  | -1.77 |  |
| STM1682 | | *tpx* | thiol peroxidase |  | -1.01 |  |
| STM1688 | | *pspC* | phage shock protein (DNA-binding transcriptional activator) |  | -1.60 | -1.38 |
| STM1689 | | *pspB* | phage shock protein |  | -1.29 | -1.33 |
| **Gene** | **Name** | | **Description** | **Log_2_FC** | | |
| **Downregulated** | | |  | **Δ*rfaL*** | **Δ*rfaG*** | **Δ*rfaD*** |
| STM1690 | | *pspA* | phage shock protein |  | -2.08 | -1.74 |
| STM1695 | | *sapD* | peptide ABC transporter ATP-binding protein |  | -1.26 |  |
| STM1712 | | *acnA* | aconitate hydratase |  | -1.70 | -1.41 |
| STM1726 | | *trpB* | tryptophan synthase subunit beta |  | -1.19 | -1.21 |
| STM1732 | | *ompW* | outer membrane protein W |  | -1.76 |  |
| STM1745 | | *oppB* | oligopeptide ABC transporter permease |  |  | -1.22 |
| STM1746.S | | *oppA* | oligopeptide ABC transporter substrate-binding protein |  |  | -1.18 |
| STM1763 | | *narH* | nitrate reductase 1 subunit beta |  | -1.09 |  |
| STM1782 | | *ychH* | inner membrane protein |  | -2.03 | -2.97 |
| STM1786 | | *-* | hydrogenase-1 small subunit |  |  | -1.76 |
| STM1787 | | *-* | hydrogenase 1 large subunit |  |  | -1.72 |
| STM1795 | | *-* | glutamate dehydrogenase |  | -2.16 |  |
| STM1798 | | *ycgR* | flagellar brake protein |  | -3.19 | -3.86 |
| STM1803 | | *dadA* | D-amino acid dehydrogenase small subunit |  | -1.87 |  |
| STM1815 | | *minD* | ATPase MinD |  | -1.17 |  |
| STM1816 | | *minE* | cell division topological specificity factor |  | -1.20 |  |
| STM1830 | | *manX* | PTS system mannose-specific transporter subunit IIAB |  |  | -1.47 |
| STM1831 | | *manY* | PTS system mannose-specific transporter subunit IIC |  | -1.47 | -2.21 |
| STM1832 | | *manZ* | PTS system mannose-specific transporter subunit IID |  | -1.50 | -2.03 |
| STM1841 | | *-* | hypothetical protein |  |  | -1.51 |
| STM1855 | | *sopE2* | guanine nucleotide exchange factor (vT3SS effector) |  | -6.47 | -8.54 |
| STM1912 | | *flhE* | flagellar protein |  | -1.69 | -2.19 |
| STM1913 | | *flhA* | flagellar biosynthesis protein |  | -1.86 | -2.21 |
| STM1914 | | *flhB* | flagellar biosynthesis protein |  | -1.28 | -1.43 |
| STM1915 | | *cheZ* | protein phosphatase (chemotaxis) |  | -3.16 | -3.36 |
| STM1916 | | *cheY* | chemotaxis regulatory protein |  | -3.39 | -4.07 |
| STM1917 | | *cheB* | chemotaxis response regulator protein-glutamate methylesterase |  | -4.36 | -5.05 |
| STM1918 | | *cheR* | chemotaxis protein methyltransferase |  | -4.15 | -6.25 |
| STM1919 | | *cheM* | methyl accepting chemotaxis protein II |  | -4.79 | -6.87 |
| STM1920 | | *cheW* | chemotaxis protein |  | -4.39 | -5.00 |
| STM1921 | | *cheA* | chemotaxis protein |  | -4.46 | -5.39 |
| STM1922 | | *motB* | motility protein B |  | -3.83 | -5.61 |
| STM1923 | | *motA* | motility protein protein A |  | -3.97 | -6.10 |
| STM1924.S | | *flhC* | transcriptional regulator (flagellar master regulatory subunit FlhC) |  | -2.75 | -3.98 |
| STM1928 | | *otsA* | alpha-trehalose-phosphate synthase | -1.04 |  |  |
| **Gene** | **Name** | | **Description** | **Log_2_FC** | | |
| **Downregulated** | | |  | **Δ*rfaL*** | **Δ*rfaG*** | **Δ*rfaD*** |
| STM1934 | | *-* | outer membrane lipoprotein |  | -1.43 | -2.49 |
| STM1950 | | *sdiA* | ftsQAZ ranscriptional regulator |  | -3.04 | -3.87 |
| STM1954 | | *fliY* | amino-acid ABC transporter substrate-binding protein |  | -1.18 | -1.26 |
| STM1955 | | *fliZ* | flagella biosynthesis protein |  | -3.47 | -3.99 |
| STM1956 | | *fliA* | RNA polymerase sigma factor |  | -3.42 | -3.85 |
| STM1958 | | *fliB* | lysine-N-methylase |  | -2.53 | -2.56 |
| STM1959 | | *fliC* | flagellin |  | -4.65 | -5.50 |
| STM1960 | | *fliD* | flagellar hook-associated protein 2 |  | -4.20 | -4.93 |
| STM1961 | | *fliS* | flagellar protein |  | -3.41 | -4.18 |
| STM1962 | | *fliT* | flagellar protein |  | -3.39 | -4.03 |
| STM1968 | | *fliE* | flagellar hook-basal body complex protein |  | -2.08 | -1.27 |
| STM1969 | | *fliF* | flagellar MS-ring protein |  | -2.10 | -2.05 |
| STM1970 | | *fliG* | flagellar motor switch protein |  | -2.67 | -2.58 |
| STM1971 | | *fliH* | flagellar assembly protein |  | -2.70 | -2.54 |
| STM1972 | | *fliI* | flagellum-specific ATP synthase |  | -2.93 | -2.91 |
| STM1973 | | *fliJ* | flagellar protein |  | -3.28 | -3.50 |
| STM1974 | | *fliK* | flagellar hook-length control protein |  | -3.59 | -3.82 |
| STM1975 | | *fliL* | flagellar basal body-associated protein |  | -2.30 | -2.50 |
| STM1976 | | *fliM* | flagellar motor switch protein |  | -2.25 | -2.72 |
| STM1977 | | *fliN* | flagellar motor switch protein |  | -2.15 | -2.58 |
| STM1978 | | *fliO* | flagellar biosynthesis protein |  | -1.75 | -2.34 |
| STM1979 | | *fliP* | flagellar biosynthesis protein |  | -1.06 | -1.71 |
| STM2016 | | *cobT* | nicotinate-nucleotide-dimethylbenzimidazole phosphoribosyltransferase |  | -1.21 |  |
| STM2018 | | *cobU* | bifunctional adenosylcobinamide kinase/adenosylcobinamide-phosphate guanylyltransferase |  | -1.45 | -1.42 |
| STM2019 | | *cbiP* | cobyric acid synthase |  | -1.23 | -1.49 |
| STM2020 | | *cbiO* | cobalt ABC transporter ATP-binding protein CbiO |  | -1.26 | -1.52 |
| STM2021 | | *cbiQ* | cobalt ABC transporter permease CbiQ |  | -1.67 | -1.87 |
| STM2022 | | *cbiN* | cobalt ABC transporter substrate-binding protein |  | -1.96 | -2.30 |
| STM2023 | | *cbiM* | cobalt ABC transporter substrate-binding protein |  | -2.06 | -2.15 |
| STM2024 | | *cbiL* | cobalt-precorrin-2 C(20)-methyltransferase |  | -1.55 | -1.99 |
| STM2025 | | *cbiK* | sirohydrochlorin cobaltochelatase |  | -1.55 | -1.82 |
| STM2027 | | *cbiH* | precorrin-3B C(17)-methyltransferase |  | -1.80 | -1.76 |
| STM2028 | | *cbiG* | cobalt-precorrin 5A hydrolase |  | -1.61 | -1.61 |
| STM2029 | | *cbiF* | precorrin-4 C11-methyltransferase |  | -1.82 | -1.85 |
| STM2030 | | *cbiT* | cobalt-precorrin-6Y C(15)-methyltransferase |  | -1.89 | -1.66 |
| **Gene** | **Name** | | **Description** | **Log_2_FC** | | |
| **Downregulated** | | |  | **Δ*rfaL*** | **Δ*rfaG*** | **Δ*rfaD*** |
| STM2031 | | *cbiE* | cobalt-precorrin-6Y C(5)-methyltransferase |  | -1.33 | -1.20 |
| STM2033 | | *cbiC* | cobalt-precorrin-8X methylmutase |  |  | -1.05 |
| STM2037 | | *pduF* | propanediol diffusion facilitator |  |  | -2.92 |
| STM2038 | | *pduA* | propanediol utilization polyhedral body protein |  | -1.66 | -3.21 |
| STM2039 | | *pudB* | propanediol utilization polyhedral body protein |  | -1.99 | -3.17 |
| STM2040 | | *pduC* | propanediol dehydratase large subunit |  | -2.74 | -3.70 |
| STM2041 | | *pduD* | propanediol dehydratase medium subunit |  | -3.06 | -3.53 |
| STM2042 | | *pduE* | propanediol dehydratase small subunit |  | -2.87 | -3.34 |
| STM2043 | | *pduG* | propanediol dehydratase reactivation protein |  | -2.18 | -2.86 |
| STM2044 | | *pduH* | propanediol dehydratase reactivation protein |  | -2.03 | -2.89 |
| STM2045 | | *pduJ* | propanediol utilization polyhedral body protein |  | -2.39 | -3.15 |
| STM2046 | | *pduK* | propanediol utilization polyhedral body protein |  | -2.22 | -2.99 |
| STM2047 | | *pduL* | phosphate propanoyltransferase |  | -2.12 | -2.52 |
| STM2048 | | *pduM* | propanediol utilization protein |  | -2.40 | -2.84 |
| STM2049 | | *pduN* | propanediol utilization polyhedral body protein |  | -1.99 | -2.19 |
| STM2050 | | *pduO* | propanediol utilization protein |  | -2.40 | -2.65 |
| STM2051 | | *pduP* | CoA-dependent propionaldehyde dehydrogenase |  | -2.66 | -2.85 |
| STM2052 | | *pduQ* | propanol dehydrogenase |  | -2.38 | -2.62 |
| STM2053 | | *pduS* | propanediol utilization polyhedral body protein |  | -2.53 | -2.41 |
| STM2054 | | *pduT* | propanediol utilization polyhedral body protein |  | -2.21 | -2.37 |
| STM2055 | | *pduU* | propanediol utilization polyhedral body protein |  | -2.60 | -2.45 |
| STM2056 | | *pduV* | propanediol utilization protein |  | -1.65 | -1.80 |
| STM2057 | | *pduW* | propionate kinase |  | -1.06 | -1.81 |
| STM2063 | | *phsC* | thiosulfate reductase cytochrome B subunit |  | -1.30 | -2.63 |
| STM2064 | | *phsB* | thiosulfate reductase electron transport protein |  | -1.71 | -2.72 |
| STM2065 | | *phsA* | thiosulfate reductase | -1.05 | -1.27 | -2.14 |
| STM2066 | | *sopA* | E3 ubiquitin-protein ligase SopA (vT3SS effector) | -1.07 | -5.56 | -6.31 |
| STM2091 | | *rfbG* | CDP glucose 4,6-dehydratase |  | -1.17 |  |
| STM2093 | | *rfbI* | CDP-6-deoxy-delta-3,4-glucoseen reductase |  | -1.28 |  |
| STM2094 | | *rfbC* | TDP-4-deoxyrhamnose 3,5 epimerase |  | -1.12 |  |
| STM2141 | | *fbaB* | fructose-bisphosphate aldolase | -1.16 |  |  |
| STM2142 | | *yegT* | MFS family transport protein |  |  | -1.10 |
| STM2152 | | *stcA* | fimbrial-like protein |  | -1.59 |  |
| **Gene** | **Name** | | **Description** | **Log_2_FC** | | |
| **Downregulated** | | |  | **Δ*rfaL*** | **Δ*rfaG*** | **Δ*rfaD*** |
| STM2176 | | *-* | glutathione S-transferase |  | -1.40 | -1.16 |
| STM2177 | | *-* | flutathione S-transferase |  | -1.59 | -1.56 |
| STM2178 | | *-* | 1,2-dioxygenase |  | -2.25 | -1.46 |
| STM2179 | | *-* | sugar transporter |  | -2.36 |  |
| STM2188 | | *mglC* | galactose/methylgalactoside ABC transporter permease |  | -1.72 |  |
| STM2189 | | *mglA* | galactose/methylgalactoside ABC transporter ATP-binding protein |  | -1.82 |  |
| STM2190 | | *mglB* | galactose/methylgalactoside ABC transporter substrate-binding protein |  | -1.12 |  |
| STM2201 | | *yeiE* | LysR family transcriptional regulator |  | -1.14 | -1.50 |
| STM2202 | | *yeiH* | inner membrane protein |  |  | -1.35 |
| STM2252 | | *ccmC* | heme exporter protein |  | -1.30 |  |
| STM2253 | | *ccmB* | heme exporter protein |  | -1.19 |  |
| STM2258 | | *napG* | ferredoxin-type protein |  | -1.44 |  |
| STM2259 | | *napA* | periplasmic nitrate reductase large subunit | -1.20 | -1.62 |  |
| STM2266 | | *apbE* | thiamine biosynthesis lipoprotein |  | -1.52 | -1.09 |
| STM2267 | | *ompC* | outer membrane porin protein C |  | -1.96 | -1.99 |
| STM2271 | | *rcsC* | sensor histidine kinase RscC |  | -1.07 |  |
| STM2275 | | *-* | GntR family regulatory protein |  | -2.65 | -2.58 |
| STM2277 | | *nrdA* | ribonucleotide-diphosphate reductase subunit alpha |  | -1.54 |  |
| STM2278 | | *nrdB* | ribonucleotide-diphosphate reductase subunit beta |  | -1.39 |  |
| STM2282 | | *glpQ* | glycerophosphodiester phosphodiesterase |  | -1.91 |  |
| STM2283 | | *glpT* | sn-glycerol-3-phosphate transporter | -1.11 | -1.11 |  |
| STM2289 | | *-* | 2-keto-3-deoxy-L-rhamnonate aldolase |  |  | -1.13 |
| STM2290 | | *yfaV* | MFS family transport protein |  |  | -1.44 |
| STM2291 | | *yfaW* | L-rhamnonate dehydratase |  | -1.36 | -1.76 |
| STM2292 | | *yfaX* | transcriptional regulator |  | -1.26 | -1.96 |
| STM2299 | | *yfbG* | bifunctional UDP-glucuronic acid decarboxylase/UDP-4-amino-4-deoxy-L-arabinose formyltransferase |  | -1.14 |  |
| STM2314 | | *-* | chemotaxis signal transduction protein (CheV) |  | -4.77 | -6.21 |
| STM2319 | | *nuoK* | NADH-quinone oxidoreductase subunit K |  | -1.08 |  |
| STM2322 | | *nuoH* | NADH-quinone oxidoreductase subunit H |  | -1.30 |  |
| STM2323.S | | *nuoG* | NADH-quinone oxidoreductase subunit G |  | -1.36 |  |
| STM2324 | | *nuoF* | NADH-quinone oxidoreductase subunit F |  | -1.83 |  |
| STM2325 | | *nuoE* | NADH-quinone oxidoreductase subunit E |  | -1.57 |  |
| STM2326 | | *nuoC* | bifunctional NADH-ubiquinone oxidoreductase subunit C/D |  | -1.54 |  |
| STM2340 | | *-* | transketolase |  | -1.65 | -1.68 |
| **Gene** | **Name** | | **Description** | **Log_2_FC** | | |
| **Downregulated** | | |  | **Δ*rfaL*** | **Δ*rfaG*** | **Δ*rfaD*** |
| STM2341 | | *-* | transketolase | -1.10 | -2.27 | -2.39 |
| STM2342 | | *ulaA* | PTS system ascorbate-specific transporter subunit IIC |  | -1.74 | -2.20 |
| STM2343 | | *-* | PTS system ascorbate-specific transporter subunit IIB |  |  | -1.99 |
| STM2344 | | *-* | PTS system transporter subunit IIA |  |  | -1.39 |
| STM2355 | | *argT* | lysine/arginine/ornithine ABC transporter substrate-binding protein |  | -1.50 |  |
| STM2359 | | *-* | amino acid transporter |  | -1.11 | -1.48 |
| STM2360 | | *-* | diaminopimelate decarboxylase |  |  | -1.99 |
| STM2391 | | *fadL* | long-chain fatty acid transporter |  | -1.22 |  |
| STM2437 | | *yfeJ* | glutamine amidotransferase | -1.72 |  |  |
| STM2457 | | *eutC* | ethanolamine ammonia-lyase small subunit |  | -1.91 | -1.65 |
| STM2458 | | *eutB* | ethanolamine ammonia-lyase large subunit |  | -1.95 | -2.05 |
| STM2461 | | *eutG* | ethanol dehydrogenase |  | -1.63 | -1.99 |
| STM2462 | | *eutJ* | ethanolamine utilization protein |  | -1.02 | -1.62 |
| STM2465 | | *eutM* | carboxysome structural protein |  |  | -1.23 |
| STM2469 | | *eutP* | ethanolamine utilization protein |  |  | -1.13 |
| STM2472 | | *maeB* | malic enzyme |  | -1.47 |  |
| STM2473 | | *talA* | transaldolase A | -1.02 | -1.21 |  |
| STM2474 | | *tktB* | transketolase |  | -1.46 |  |
| STM2526 | | *ndk* | nucleoside diphosphate kinase |  | -1.12 |  |
| STM2535 | | *sseB* | enhanced serine sensitivity protein (vT3SS effector) |  | -1.05 |  |
| STM2548 | | *asrA* | anaerobic sulfite reductase subunit A |  |  | -1.71 |
| STM2549 | | *asrB* | anaerobic sulfite reductase subunit B |  |  | -2.16 |
| STM2550 | | *asrC* | anaerobic sulfite reductase subunit C |  |  | -1.44 |
| STM2556 | | *hmpA* | bifunctional nitric oxide dioxygenase/dihydropteridine reductase |  | -1.36 |  |
| STM2559 | | *cadA* | lysine decarboxylase 1 |  |  | -1.03 |
| STM2605 | | *-* | head-tail preconnector-like protein |  | -1.18 | -1.33 |
| STM2629 | | *-* | hypothetical protein |  |  | -2.34 |
| STM2630 | | *-* | hypothetical protein |  | -1.73 | -2.49 |
| STM2631 | | *-* | hypothetical protein |  | -2.13 | -3.20 |
| STM2632 | | *-* | exodeoxyribonuclease VIII-like protein |  | -1.82 | -2.20 |
| STM2633.S | | *-* | enterohemolysin 1-like protein |  | -2.19 | -2.23 |
| STM2634 | | *-* | hypothetical protein |  | -2.48 | -1.94 |
| STM2643 | | *srmB* | ATP-dependent RNA helicase |  | -1.04 | -1.01 |
| STM2660 | | *clpB* | chaperone protein ClpB |  | -1.04 |  |
| STM2720 | | *-* | major capsid-like protein |  |  | -2.17 |
| STM2770 | | *fljA* | phase-1 flagellin repressor |  | -4.35 | -6.31 |
| **Gene** | **Name** | | **Description** | **Log_2_FC** | | |
| **Downregulated** | | |  | **Δ*rfaL*** | **Δ*rfaG*** | **Δ*rfaD*** |
| STM2771 | | *fljB* | phase 2 flagellin |  | -6.03 | -9.06 |
| STM2777 | | *iroN* | iron-enterobactin outer membrane transporter (FepA) |  |  | -1.26 |
| STM2785 | | *tctD* | transcriptional regulator |  | -1.26 |  |
| STM2790 | | *ygaF* | hydroxyglutarate oxidase |  | -1.61 |  |
| STM2797 | | *-* | ArsR family regulatory protein |  |  | -1.18 |
| STM2833 | | *srlE* | PTS system glucitol/sorbitol-specific transporter subunit IICB |  |  | -1.88 |
| STM2834 | | *slrB* | PTS system glucitol/sorbitol-specific transporter subunit IIA | -1.24 | -1.16 | -1.85 |
| STM2835 | | *srlD* | glucitol/sorbitol-6-phosphate dehydrogenase |  |  | -1.47 |
| STM2865 | | *avrA* | secreted effector protein | -1.03 | -3.12 | -3.64 |
| STM2866 | | *sprB* | transcriptional regulator | -1.14 | -4.40 | -4.56 |
| STM2867 | | *hilC* | transcriptional regulator |  | -1.24 | -1.95 |
| STM2868 | | *orgC* | cytoplasmic protein |  | -3.12 | -2.37 |
| STM2869 | | *orgB* | oxygen-regulated invasion protein |  | -3.43 | -2.60 |
| STM2870 | | *orgA* | oxygen-regulated invasion protein |  | -4.60 | -4.31 |
| STM2871 | | *prgK* | secretion system lipoprotein |  | -4.91 | -5.57 |
| STM2872 | | *prgJ* | secretion system protein |  | -5.70 | -7.58 |
| STM2873 | | *prgI* | secretion system protein |  | -5.82 | -7.61 |
| STM2874 | | *prgH* | secretion system protein |  | -5.85 | -8.80 |
| STM2875 | | *hilD* | transcriptional regulator |  | -3.29 | -3.44 |
| STM2876 | | *hilA* | transcriptional regulator |  | -5.90 | -7.73 |
| STM2877 | | *iagB* | invasion protein |  | -5.00 | -6.47 |
| STM2878 | | *sptP* | secreted effector protein |  | -4.50 | -3.98 |
| STM2879 | | *sicP* | chaperone protein |  | -5.15 | -4.10 |
| STM2881 | | *iacP* | acyl carrier protein | -1.25 | -4.72 | -4.23 |
| STM2882 | | *sipA* | cell invasion protein | -1.38 | -7.85 | -8.21 |
| STM2883 | | *sipD* | cell invasion protein | -1.32 | -8.73 | -9.25 |
| STM2884 | | *sipC* | cell invasion protein | -1.34 | -7.83 | -8.49 |
| STM2885 | | *sipB* | cell invasion protein | -1.10 | -7.98 | -8.61 |
| STM2886 | | *sicA* | chaperone protein |  | -7.43 | -8.39 |
| STM2887 | | *spaS* | surface presentation of antigens protein |  | -6.79 | -7.04 |
| STM2888 | | *spaR* | surface presentation of antigens protein |  | -4.86 | -7.25 |
| STM2889 | | *spaQ* | surface presentation of antigens protein |  | -5.77 | -6.85 |
| STM2890 | | *spaP* | surface presentation of antigens protein |  | -6.02 | -7.53 |
| STM2891 | | *spaO* | surface presentation of antigens protein | -1.05 | -7.01 | -8.11 |
| STM2892 | | *invJ* | surface presentation of antigens protein |  | -7.47 | -9.44 |
| STM2893 | | *invI* | surface presentation of antigens protein | -1.45 | -6.90 | -9.80 |
| STM2894 | | *invC* | ATP synthase (SpaL) |  | -6.30 | -8.53 |
|  | |  |  |  |  |  |
| **Gene** | **Name** | | **Description** | **Log_2_FC** | | |
| **Downregulated** | | |  | **Δ*rfaL*** | **Δ*rfaG*** | **Δ*rfaD*** |
| STM2895 | | *invB* | surface presentation of antigens protein (SpaK) | -1.38 | -6.58 | -9.15 |
| STM2896 | | *invA* | invasion protein |  | -6.10 | -8.91 |
| STM2897 | | *invE* | invasion protein |  | -5.41 | -8.31 |
| STM2898 | | *invG* | invasion protein |  | -5.21 | -8.98 |
| STM2899 | | *invF* | transcriptional regulator |  | -5.44 | -9.11 |
| STM2900 | | *invH* | invasion lipoprotein | -1.08 | -3.72 | -5.53 |
| STM2940 | | *-* | cytoplasmic protein |  | -1.91 |  |
| STM2941 | | *yghJ* | cytoplasmic protein |  | -1.62 | -1.38 |
| STM2943 | | *-* | cytoplasmic protein |  | -1.28 | -1.22 |
| STM2945 | | *sopD* | secreted effector protein (vT3SS) |  | -5.05 | -6.07 |
| STM2952 | | *eno* | enolase | -1.04 |  |  |
| STM3071 | | *-* | DNA-binding protein | -2.04 |  |  |
| STM2976 | | *fucI* | L-fucose isomerase |  | -2.05 | -2.09 |
| STM2977 | | *fucK* | L-fuculokinase |  | -1.42 | -1.26 |
| STM3138 | | *-* | methyl-accepting chemotaxis protein |  | -5.12 | -5.97 |
| STM3151 | | *yghW* | cytoplasmic protein |  |  | -2.16 |
| STM3152 | | *-* | methyl-accepting chemotaxis protein |  | -2.96 | -3.56 |
| STM3154 | | *-* | ATP-dependent RNA helicase-like protein |  | -2.25 | -3.29 |
| STM3155 | | *-* | cytoplasmic protein |  | -2.81 | -3.70 |
| STM3156 | | *-* | cytoplasmic protein |  | -3.62 | -3.93 |
| STM3164 | | *yqhD* | alcohol dehydrogenase |  | -1.09 | -1.26 |
| STM3168 | | *ygiR* | hypothetical protein |  | -1.10 |  |
| STM3169 | | *-* | periplasmic dicarboxylate-binding protein |  | -1.66 | -2.67 |
| STM3170 | | *-* | inner membrane protein |  |  | -3.19 |
| STM3216 | | *-* | methyl-accepting chemotaxis protein |  | -4.75 | -5.52 |
| STM3217 | | *aer* | aerotaxis sensor receptor |  | -4.25 | -4.93 |
| STM3238 | | *yhaN* | hypothetical protein |  |  | -1.64 |
| STM3239 | | *yhaO* | HAAAP family transport protein |  |  | -2.50 |
| STM3240 | | *tdcG* | L-serine deaminase | -1.23 | -1.88 | -3.67 |
| STM3241 | | *tdcE* | pyruvate formate-lyase 4/2-ketobutyrate formate-lyase | -2.20 | -2.41 | -3.71 |
| STM3242 | | *tdcD* | propionate/acetate kinase | -3.40 | -1.61 | -3.53 |
| STM3243 | | *tdcC* | threonine/serine transporter TdcC | -2.94 |  | -3.24 |
| STM3244 | | *tdcB* | threonine dehydratase TdcB | -3.18 |  | -1.75 |
| STM3245 | | *tdcA* | DNA-binding transcriptional activator TdcA | -1.07 |  |  |
| STM3255 | | *-* | PTS system fructose-specific transporter subunit IIB |  | -1.08 | -1.24 |
| STM3280.S | | *deaD* | ATP-dependent RNA helicase |  | -1.24 | -1.37 |
| STM3286 | | *infB* | translation initiation factor IF-2 |  | -1.01 |  |
| STM3291 | | *-* | cytoplasmic protein |  | -1.50 | -1.77 |
| **Gene** | **Name** | | **Description** | **Log_2_FC** | | |
| **Downregulated** | | |  | **Δ*rfaL*** | **Δ*rfaG*** | **Δ*rfaD*** |
| STM3323 | | *yhbJ* | hypothetical protein |  | -1.09 |  |
| STM3326 | | *mtgA* | monofunctional biosynthetic peptidoglycan transglycosylase |  | -1.22 | -1.15 |
| STM3327 | | *yhbL* | isoprenoid biosynthesis protein |  | -1.39 | -1.23 |
| STM3330 | | *gltB* | glutamate synthase subunit alpha |  | -1.05 |  |
| STM3359 | | *mdh* | malate dehydrogenase |  | -1.68 |  |
| STM3375 | | *yhdA* | regulatory protein (CsrD) |  | -1.67 | -2.02 |
| STM3421 | | *rplO* | 50S ribosomal protein L15 |  | -1.01 |  |
| STM3423 | | *rpsE* | 30S ribosomal protein S5 |  | -1.24 |  |
| STM3426 | | *rpsH* | 30S ribosomal protein S8 |  | -1.26 |  |
| STM3437 | | *rplB* | 50S ribosomal protein L2 | -1.03 |  |  |
| STM3438 | | *rplW* | 50S ribosomal protein L23 | -1.06 |  |  |
| STM3448 | | *rpsL* | 30S ribosomal protein S12 | -1.26 | -1.04 |  |
| STM3500 | | *pckA* | phosphoenolpyruvate carboxykinase |  | -1.30 | -1.62 |
| STM3504 | | *yhgF* | RNase R |  | -1.18 |  |
| STM3505 | | *feoA* | ferrous iron transport protein A |  | -1.56 | -1.77 |
| STM3506 | | *feoB* | ferrous iron transport protein B |  |  | -1.46 |
| STM3531 | | *-* | dihydroxyacid dehydratase |  | -1.40 | -1.52 |
| STM3536 | | *glgC* | glucose-1-phosphate adenylyltransferase |  |  | -1.23 |
| STM3548 | | *-* | cytoplasmic protein |  | -1.37 | -2.00 |
| STM3552 | | *yhhA* | outer membrane protein |  |  | -1.32 |
| STM3557 | | *ugpB* | glycerol-3-phosphate ABC transporter substrate-binding protein |  | -1.05 |  |
| STM3577 | | *tcp* | methyl-accepting transmembrane citrate/phenol chemoreceptor |  | -4.43 | -6.47 |
| STM3585 | | *yhhJ* | ABC transport ATP-binding protein |  |  | -1.20 |
| STM3586.S | | *yhiH* | multidrug ABC transporter ATPase |  |  | -1.10 |
| STM3590 | | *uspB* | universal stress protein B |  | -1.04 | -1.04 |
| STM3591 | | *uspA* | universal stress protein A |  |  | -1.23 |
| STM3600 | | *-* | sugar kinase | -1.18 |  |  |
| STM3604 | | *-* | inner membrane protein |  | -2.52 | -2.43 |
| STM3611 | | *yhjH* | diguanylate cyclase/phosphodiesterase domain-containing protein (motility-promoting) |  | -4.14 | -6.21 |
| STM3614 | | *dctA* | C4-dicarboxylate transporter |  | -1.89 |  |
| STM3618 | | *yhjN* | cellulose synthase regulator protein |  | -1.25 |  |
| STM3673 | | *yiaO* | periplasmic dicarboxylate-binding protein |  | -4.76 |  |
| STM3680 | | *aldB* | aldehyde dehydrogenase B |  | -1.13 | -1.32 |
| STM3710 | | *rfaD* | ADP-L-glycero-D-manno-heptose-6-epimerase |  |  | -3.17 |
| STM3713 | | *rfaL* | O-antigen ligase | -4.21 |  |  |
| STM3782 | | *-* | PTS system mannitol/fructose-specific transporter subunit IIC |  | -1.20 | -2.01 |
| **Gene** | **Name** | | **Description** | **Log_2_FC** | | |
| **Downregulated** | | |  | **Δ*rfaL*** | **Δ*rfaG*** | **Δ*rfaD*** |
| STM3783 | | *-* | PTS system mannitol/fructose-specific transporter subunit IIB |  |  | -1.42 |
| STM3791 | | *-* | cytoplasmic protein |  | -1.86 | -1.42 |
| STM3792 | | *-* | L-fucose permease |  | -1.21 | -1.33 |
| STM3796 | | *ilvB* | acetolactate synthase catalytic subunit |  | -1.33 | -1.02 |
| STM3809.S | | *ibpA* | heat shock protein IbpA |  | -1.37 |  |
| STM3812 | | *ccmH* | heme lyase subunit |  | -1.03 |  |
| STM3813 | | *ccmG* | cytochrome c biogenesis protein |  | -1.22 |  |
| STM3815 | | *ccmE* | cytochrome c-type biogenesis protein |  | -1.11 |  |
| STM3817 | | *ccmC* | heme exporter protein |  | -1.41 |  |
| STM3820 | | *-* | cytochrome c peroxidase |  | -2.79 | -2.40 |
| STM3823 | | *torC* | trimethylamine N-oxide reductase cytochrome c-like subunit |  | -1.42 | -1.76 |
| STM3829 | | *dgoK* | 2-oxo-3-deoxygalactonate kinase |  |  | -1.14 |
| STM3833 | | *-* | mandelate racemase |  |  | -1.28 |
| STM3865 | | *atpD* | F0F1 ATP synthase subunit beta |  | -1.02 |  |
| STM3866 | | *atpG* | F0F1 ATP synthase subunit gamma |  | -1.11 |  |
| STM3867 | | *atpA* | F0F1 ATP synthase subunit alpha |  | -1.22 |  |
| STM3877 | | *asnA* | aspartate--ammonia ligase |  |  | -1.35 |
| STM4007 | | *glnA* | glutamine synthetase |  | -1.67 | -2.42 |
| STM4020.S | | *yihR* | aldose-1-epimerase |  | -1.83 |  |
| STM4036 | | *fdoH* | formate dehydrogenase-O Fe-S subunit |  | -2.08 |  |
| STM4037 | | *fdoG* | formate dehydrogenase large subunit |  | -2.53 | -1.19 |
| STM4044 | | *-* | alcohol dehydrogenase |  |  | -1.23 |
| STM4071 | | *-* | mannose-6-phosphate isomerase |  | -1.53 | -1.74 |
| STM4072 | | *ydeV* | autoinducer-2 kinase( LsrK) |  | -1.20 | -1.60 |
| STM4074 | | *ego* | autoinducer 2 import system ATP-binding protein (LsrA) |  |  | -1.48 |
| STM4075 | | *ydeY* | autoinducer 2 import system permease (LsrC) |  | -1.11 | -2.22 |
| STM4076 | | *ydeZ* | autoinducer 2 import system permease (LsrD) |  | -2.04 | -2.61 |
| STM4077 | | *yneA* | autoinducer 2 import system substrate-binding protein (LsrB) | -1.14 | -2.69 | -2.65 |
| STM4078 | | *yneB* | autoinducer-2 aldolase (LsrF) | -1.14 | -2.60 | -2.30 |
| STM4079.S | | *yneC* | autoinducer-2 degrading protein (LsrG) |  | -2.40 | -2.36 |
| STM4080 | | *-* | epimerase |  | -1.98 | -1.92 |
| STM4086 | | *glpK* | glycerol kinase |  | -1.30 | -1.12 |
| STM4087 | | *glpF* | glycerol diffusion protein | -1.02 |  |  |
| STM4092 | | *hslV* | ATP-dependent protease peptidase subunit | -1.24 |  |  |
| STM4106 | | *katG* | catalase-peroxidase |  | -1.46 | -1.49 |
| STM4109 | | *talC* | fructose-6-phosphate aldolase |  |  | -1.04 |
|  | |  |  |  |  |  |
| **Gene** | **Name** | | **Description** | **Log_2_FC** | | |
| **Downregulated** | | |  | **Δ*rfaL*** | **Δ*rfaG*** | **Δ*rfaD*** |
| STM4112 | | *frwC* | PTS system fructose-like transporter subunit IIC |  |  | -1.04 |
| STM4126 | | *udhA* | soluble pyridine nucleotide transhydrogenase |  | -1.78 | -1.26 |
| STM4153 | | *rpoB* | DNA-directed RNA polymerase subunit beta |  | -1.89 |  |
| STM4154 | | *rpoC* | DNA-directed RNA polymerase subunit beta |  | -1.49 |  |
| STM4161 | | *-* | sulfur carrier protein (ThiS) |  | -2.96 | -2.77 |
| STM4172 | | *zraP* | zinc resistance protein |  |  | -2.48 |
| STM4239 | | *-* | cytoplasmic protein |  |  | -1.29 |
| STM4240 | | *yjbJ* | stress-response protein |  |  | -1.41 |
| STM4257 | | *-* | hypothetical protein | -1.28 | -7.87 | -9.63 |
| STM4258 | | *-* | methyl-accepting chemotaxis protein | -1.20 | -8.24 | -9.31 |
| STM4259 | | *-* | ABC exporter outer membrane protein | -1.68 | -7.98 | -9.76 |
| STM4260 | | *-* | membrane permease | -1.27 | -8.41 | -9.40 |
| STM4261 | | *-* | inner membrane protein | -1.54 | -4.76 | -4.78 |
| STM4262 | | *-* | bacteriocin/lantibiotic ABC transporter |  | -4.46 | -5.23 |
| STM4264 | | *yjcC* | diguanylate cyclase/phosphodiesterase |  |  | -1.43 |
| STM4275 | | *acs* | acetyl-CoA synthetase |  | -1.98 | -1.50 |
| STM4277 | | *nrfA* | cytochrome c552 |  |  | -1.01 |
| STM4278.S | | *nrfB* | cytochrome c nitrite reductase pentaheme subunit |  |  | -1.12 |
| STM4279 | | *nrfC* | formate-dependent nitrite reductase |  | -1.15 |  |
| STM4281 | | *nrfE* | formate-dependent nitrite reductase |  | -1.07 |  |
| STM4295 | | *adiY* | transcriptional activator | -1.57 |  |  |
| STM4300 | | *fumB* | fumarase B |  |  | -1.78 |
| STM4301 | | *dcuB* | anaerobic C4-dicarboxylate transporter |  |  | -1.41 |
| STM4306 | | *-* | anaerobic dimethylsulfoxide reductase subunit B |  |  | -1.32 |
| STM4308 | | *-* | anaerobic dehydrogenase subunit |  |  | -1.11 |
| STM4310 | | *-* | inner membrane protein |  | -1.65 | -1.54 |
| STM4312 | | *-* | hypothetical protein |  | -4.29 |  |
| STM4313 | | *-* | cytoplasmic protein |  | -2.97 | -2.96 |
| STM4314 | | *-* | LuxR family transcriptional regulator | -1.16 | -4.37 | -6.80 |
| STM4315 | | *-* | AraC-type DNA-binding domain-containing protein |  | -5.82 | -7.33 |
| STM4325 | | *dcuA* | anaerobic C4-dicarboxylate transporter |  | -1.46 | -2.30 |
| STM4329 | | *groES* | co-chaperonin | -1.88 |  |  |
| STM4330 | | *groEL* | chaperonin | -1.40 |  |  |
| STM4392 | | *priB* | primosomal replication protein N | -1.10 |  |  |
| STM4419 | | *-* | sugar transporter |  | -1.50 | -1.46 |
| STM4420 | | *-* | inner membrane protein |  |  | -1.05 |
|  | |  |  |  |  |  |
| **Gene** | **Name** | | **Description** | **Log_2_FC** | | |
| **Downregulated** | | |  | **Δ*rfaL*** | **Δ*rfaG*** | **Δ*rfaD*** |
| STM4423 | | *-* | AraC-type DNA-binding domain-containing protein |  | -1.27 | -1.91 |
| STM4424.S | | *-* | inosose dehydratase |  | -3.35 | -3.55 |
| STM4425 | | *-* | inositol 2-dehydrogenase |  | -2.51 | -3.01 |
| STM4435 | | *-* | cytoplasmic protein |  | -1.93 | -2.29 |
| STM4436 | | *-* | endonuclease |  |  | -1.40 |
| STM4443 | | *-* | inner membrane protein |  | -1.28 | -1.46 |
| STM4459 | | *pyrI* | aspartate carbamoyltransferase regulatory subunit |  |  | -1.02 |
| STM4460 | | *pyrB* | aspartate carbamoyltransferase catalytic subunit | -1.21 | -1.48 | -1.18 |
| STM4465 | | *-* | ornithine carbamoyltransferase |  | -2.43 | -2.27 |
| STM4466 | | *-* | carbamate kinase |  | -2.25 | -2.77 |
| STM4467 | | *-* | arginine deiminase |  | -1.88 | -2.61 |
| STM4481 | | *idnR* | L-idonate regulator |  |  | -1.15 |
| STM4495 | | *-* | type II restriction enzyme methylase subunit |  | -1.12 |  |
| STM4510 | | *-* | aspartate racemase |  | -1.97 | -5.32 |
| STM4511 | | *yjiE* | LysR family transcriptional regulator |  | -1.85 | -3.95 |
| STM4512 | | *iadA* | isoaspartyl dipeptidase |  | -2.83 | -4.98 |
| STM4513 | | *yjiG* | inner membrane protein |  | -2.12 | -5.06 |
| STM4514.S | | *yjiH* | inner membrane protein |  | -1.38 | -5.52 |
| STM4519 | | *-* | NAD-dependent aldehyde dehydrogenase | -1.22 |  |  |
| STM4533 | | *tsr* | methyl-accepting chemotaxis protein I |  | -3.32 | -4.43 |
| STM4569 | | *deoB* | phosphopentomutase |  | -1.15 |  |
| STM4570 | | *deoD* | purine nucleoside phosphorylase |  | -1.41 |  |
| **Gene** | **Name** | | **Description** | **Log_2_FC** | | |
| **Upregulated** | | |  | **Δ*rfaL*** | **Δ*rfaG*** | **Δ*rfaD*** |
| STM0001 | | *thrL* | thr operon leader peptide | 1.42 |  |  |
| STM0002 | | *thrA* | bifunctional aspartokinase I/homoserine dehydrogenase I | 1.00 |  |  |
| STM0004 | | *thrC* | threonine synthase | 1.02 |  |  |
| STM0054 | | *-* | oxalacetate decarboxylase subunit beta |  |  | 2.37 |
| STM0055 | | *-* | oxaloacetate decarboxylase subunit alpha |  |  | 3.47 |
| STM0056 | | *-* | oxaloacetate decarboxylase subunit gamma |  |  | 999.00 |
| STM0057 | | *-* | citrate-sodium symporter |  |  | 4.54 |
| STM0058 | | *citC2* | citrate lyase synthetase |  |  | 6.00 |
| STM0059 | | *citD2* | citrate lyase subunit gamma |  |  | 4.20 |
| STM0060 | | *citE2* | citrate lyase subunit beta |  |  | 4.77 |
| STM0061 | | *citF2* | citrate lyase subunit alpha |  |  | 4.27 |
| STM0062 | | *citX2* | cytoplasmic protein |  |  | 3.51 |
| **Gene** | **Name** | | **Description** | **Log_2_FC** | | |
| **Upregulated** | | |  | **Δ*rfaL*** | **Δ*rfaG*** | **Δ*rfaD*** |
| STM0063 | | *citG2* | 2-(5''-triphosphoribosyl)-3'-dephosphocoenzyme-A synthase |  |  | 3.40 |
| STM0064 | | *dapB* | 4-hydroxy-tetrahydrodipicolinate reductase |  |  | 1.01 |
| STM0080 | | *-* | outer membrane lipoprotein |  | 2.68 |  |
| STM0089 | | *apaG* | protein |  | 1.27 | 1.12 |
| STM0114 | | *leuL* | leu operon leader peptide | 3.46 | 3.41 | 2.75 |
| STM0119 | | *yabB* | cell division protein (MraZ) |  | 1.06 |  |
| STM0122 | | *ftsI* | peptidoglycan synthase | 1.74 |  |  |
| STM0138 | | *yacG* | DNA gyrase inhibitor |  | 1.16 |  |
| STM0164 | | *-* | LysR family transcriptional regulator | 1.40 |  |  |
| STM0165 | | *speD* | S-adenosylmethionine decarboxylase |  |  | 1.51 |
| STM0167 | | *yacC* | periplasmic protein |  | 1.66 | 1.98 |
| STM0179 | | *yadE* | xylanase/chitin deacetylase | 1.18 | 1.64 | 1.20 |
| STM0186 | | *dksA* | RNA polymerase-binding transcription factor |  |  | 1.15 |
| STM0187 | | *sfsA* | sugar fermentation stimulation protein A |  | 1.10 |  |
| STM0209 | | *htrA* | serine endoprotease |  |  | 2.48 |
| STM0210 | | *cdaR* | carbohydrate diacid transcriptional activator |  |  | 1.78 |
| STM0216 | | *rpsB* | 30S ribosomal protein S2 |  |  | 1.87 |
| STM0217 | | *tsf* | elongation factor Ts |  |  | 1.89 |
| STM0220 | | *dxr* | 1-deoxy-D-xylulose 5-phosphate reductoisomerase | 1.06 | 1.67 |  |
| STM0225 | | *hlpA* | chaperone protein (Skp) |  |  | 1.08 |
| STM0226 | | *lpxD* | UDP-3-O-[3-hydroxymyristoyl] glucosamine N-acyltransferase |  |  | 1.23 |
| STM0229 | | *lpxB* | lipid-A-disaccharide synthase | 2.13 | 1.90 | 1.35 |
| STM0230 | | *rnhB* | ribonuclease HII |  | 1.00 | 1.03 |
| STM0240 | | *yaeJ* | hydrolase domain-containing protein |  | 1.27 |  |
| STM0263 | | *rnhA* | ribonuclease H |  | 1.42 | 1.48 |
| STM0264 | | *dnaQ* | DNA polymerase III subunit epsilon | 1.28 | 1.26 |  |
| STM0277 | | *-* | cytoplasmic protein |  | 1.49 | 1.12 |
| STM0307 | | *-* | VirG-like protein |  | 1.49 | 1.49 |
| STM0309 | | *fadE* | acyl-CoA dehydrogenase |  | 1.04 |  |
| STM0321 | | *proB* | glutamate 5-kinase |  |  | 1.20 |
| STM0327 | | *-* | cytoplasmic protein |  | 1.30 |  |
| STM0349 | | *-* | outer membrane lipoprotein |  |  | 1.06 |
| STM0365 | | *yahN* | transporter |  | 1.29 |  |
| STM0374 | | *yaiV* | DNA-binding transcriptional regulator |  | 1.36 |  |
| STM0375 | | *ampH* | penicillin-binding protein |  | 1.34 |  |
| STM0378 | | *yaiY* | inner membrane protein |  | 1.51 |  |
| STM0383 | | *yaiB* | anti-adapter protein (IraP) |  | 1.07 | 1.08 |
| STM0401 | | *malZ* | maltodextrin glucosidase |  | 2.58 | 3.46 |
| **Gene** | **Name** | | **Description** | **Log_2_FC** | | |
| **Upregulated** | | |  | **Δ*rfaL*** | **Δ*rfaG*** | **Δ*rfaD*** |
| STM0406 | | *yajC* | preprotein translocase membrane subunit |  |  | 1.27 |
| STM0447 | | *tig* | trigger factor |  |  | 1.55 |
| STM0461 | | *mdlB* | multidrud ABC transporter permease/ATP-binding protein |  | 1.00 |  |
| STM0468 | | *ylaB* | diguanylate cyclase/phosphodiesterase domain-containing protein | 1.12 | 1.20 |  |
| STM0470 | | *rpmJ* | 50S ribosomal protein L36 | 2.00 | 1.30 |  |
| STM0472 | | *maa* | maltose O-acetyltransferase |  | 1.15 |  |
| STM0473 | | *hha* | hemolysin expression-modulating protein |  | 1.20 |  |
| STM0484 | | *dnaX* | DNA polymerase III subunits gamma and tau |  |  | 1.11 |
| STM0486 | | *recR* | recombination protein |  |  | 1.11 |
| STM0474 | | *ybaJ* | cytoplasmic protein |  | 1.12 |  |
| STM0536 | | *ppiB* | peptidyl-prolyl cis-trans isomerase |  |  | 1.05 |
| STM05680 | | *-* | hypothetical protein | 1.03 | 2.30 | 1.10 |
| STM0581 | | *-* | regulatory protein |  |  | 1.09 |
| STM0589 | | *fepE* | ferric enterobactin transport protein |  |  | 1.02 |
| STM0617 | | *rna* | RNase I |  | 1.24 |  |
| STM0618 | | *citT* | citrate/succinate transporter |  |  | 3.49 |
| STM0619 | | *citG* | 2-(5''-triphosphoribosyl)-3'-dephospho-CoA synthase |  |  | 7.05 |
| STM0620 | | *citX* | apo-citrate lyase phosphoribosyl-dephospho-CoA transferase |  |  | 7.69 |
| STM0621 | | *citF* | citrate lyase subunit alpha/citrate-ACP transferase |  |  | 6.42 |
| STM0622 | | *citE* | citrate lyase subunit beta |  |  | 6.06 |
| STM0623 | | *citD* | citrate lyase subunit gamma |  |  | 7.24 |
| STM0624 | | *citC* | citrate lyase synthetase |  | 3.35 | 8.04 |
| STM0625 | | *dpiB* | sensory histidine kinase |  | 1.72 | 2.11 |
| STM0626 | | *dpiA* | two-component response regulator DpiA |  | 1.68 | 2.31 |
| STM0628 | | *pagP* | lipid A palmitoyltransferase |  | 1.31 | 1.24 |
| STM0629 | | *cspE* | RNA chaperone | 1.63 | 1.25 |  |
| STM0632 | | *tatE* | Sec-independent protein translocase protein |  | 1.02 |  |
| STM0635.S | | *lipB* | octanoyltransferase |  | 1.46 | 1.92 |
| STM0636 | | *ybeD* | hypothetical protein | 1.43 |  |  |
| STM0651 | | *-* | 2-keto-3-deoxygluconate permease | 1.40 | 1.23 |  |
| STM0681 | | *nagD* | UMP phosphatase |  | 1.01 |  |
| STM0693 | | *fur* | ferric uptake regulator |  | 1.20 | 1.13 |
| STM0708 | | *ybfA* | periplasmic protein | 2.14 |  |  |
| STM0771 | | *-* | cobalamin/Fe3+-siderophore ABC transporter ATP-binding protein |  | 1.72 |  |
| STM0792 | | *ybhB* | kinase inhibitor protein |  | 1.84 | 1.67 |
| STM0814 | | *ybhQ* | inner membrane protein |  | 1.43 | 1.41 |
| **Gene** | **Name** | | **Description** | **Log_2_FC** | | |
| **Upregulated** | | |  | **Δ*rfaL*** | **Δ*rfaG*** | **Δ*rfaD*** |
| STM0860 | | *-* | inner membrane protein |  | 1.35 |  |
| STM0865 | | *ybjG* | undecaprenyl pyrophosphate phosphatase |  | 1.38 |  |
| STM0870 | | *-* | transport protein |  | 1.20 |  |
| STM0871 | | *ybjM* | inner membrane protein |  | 1.43 |  |
| STM0881 | | *ybjO* | inner membrane protein |  | 1.11 |  |
| STM0904 | | *-* | hypothetical protein |  | 1.50 |  |
| STM0909 | | *-* | hypothetical protein |  |  | 1.39 |
| STM0916 | | *-* | phage major tail protein |  |  | 1.16 |
| STM0924 | | *-* | Cu-Zn superoxide dismutase |  | 1.85 | 1.47 |
| STM0926 | | *-* | minor tail protein | 1.26 |  | 1.49 |
| STM0996 | | *ycbK* | outer membrane protein |  |  | 1.33 |
| STM0997 | | *ycbL* | metallo-beta-lactamase |  |  | 1.37 |
| STM1017 | | *-* | hypothetical protein |  | 1.24 |  |
| STM1023 | | *-* | hypothetical protein | 1.24 |  |  |
| STM1062 | | *uup* | ABC transporter ATPase |  |  | 1.16 |
| STM1066 | | *rmf* | ribosome modulation factor | 1.14 |  |  |
| STM1084 | | *yccK* | sulfurtransferase (TusE) |  |  | 1.13 |
| STM1093 | | *-* | hypothetical protein (SPI-5) |  | 2.53 |  |
| STM1096 | | *copR* | copper resistance transcriptional regulator |  | 1.10 |  |
| STM1121 | | *ymdF* | cytoplasmic protein | 1.54 | 2.34 |  |
| STM1149 | | *mdoC* | glucans biosynthesis protein C |  | 1.25 |  |
| STM1155 | | *htrB* | lipid A biosynthesis lauroyl acyltransferase |  | 1.34 | 1.06 |
| STM1166 | | *yceL* | multidrug resistance protein (MdtH) |  | 1.30 |  |
| STM1195 | | *fabG* | 3-oxoacyl-ACP reductase |  |  | 1.32 |
| STM1196 | | *acpP* | acyl carrier protein |  | 1.05 |  |
| STM1212 | | *ycfJ* | outer membrane lipoprotein |  | 1.33 |  |
| STM1219 | | *ycfW* | outer membrane-specific lipoprotein transporter subunit (LolD) | 1.01 |  |  |
| STM1228 | | *-* | periplasmic protein |  | 1.11 |  |
| STM1254 | | *-* | outer membrane lipoprotein |  | 2.72 | 2.96 |
| STM1257 | | *-* | ABC transporter permease |  |  | 1.58 |
| STM1258 | | *-* | ABC transporter ATP-binding protein |  |  | 1.23 |
| STM1261 | | *-* | cytoplasmic protein |  | 1.37 | 1.61 |
| STM1266 | | *-* | transcriptional regulator |  | 1.25 | 1.25 |
| STM1267 | | *-* | cytoplasmic protein |  |  | 1.04 |
| STM1273 | | *-* | nitric oxide reductase |  | 1.19 |  |
| STM1292 | | *yeaC* | cytoplasmic protein | 1.07 |  |  |
| STM1308 | | *spy* | stress response protein |  |  | 1.10 |
| STM1343 | | *nlpC* | lipoprotein |  | 1.90 | 1.73 |
| STM1344 | | *ydiV* | anti-FlhC(2)FlhD(4) factor |  | 1.59 |  |
| **Gene** | **Name** | | **Description** | **Log_2_FC** | | |
| **Upregulated** | | |  | **Δ*rfaL*** | **Δ*rfaG*** | **Δ*rfaD*** |
| STM1347 | | *aroH* | phospho-2-dehydro-3-deoxyheptonate aldolase | 1.05 | 1.14 |  |
| STM1362 | | *ydiL* | cytoplasmic protein |  | 1.47 | 1.85 |
| STM1475 | | *rstA* | response regulator |  | 1.64 | 1.91 |
| STM1377 | | *lpp* | major outer membrane lipoprotein | 1.64 |  |  |
| STM1519.S | | *marA* | DNA-binding transcriptional activator |  | 1.12 |  |
| STM1520 | | *marR* | DNA-binding transcriptional repressor |  | 1.26 |  |
| STM1521 | | *marC* | inner membrane protein |  | 1.10 |  |
| STM1564 | | *yddX* | biofilm-dependent modulation protein | 1.04 | 2.68 |  |
| STM1603 | | *yncJ* | periplasmic protein | 1.53 |  |  |
| STM1648 | | *hslJ* | heat-inducible protein HslJ |  | 1.61 |  |
| STM1686 | | *pspE* | thiosulfate:cyanide sulfurtransferase (phage shock protein E) |  | 1.31 |  |
| STM1698 | | *-* | secreted effector kinase (SteC) |  | 1.48 |  |
| STM1700 | | *fabI* | enoyl-ACP reductase |  |  | 1.04 |
| STM1701 | | *yciW* | cytoplasmic protein |  | 1.31 |  |
| STM1705 | | *osmB* | osmotically inducible lipoprotein B |  | 1.31 |  |
| STM1715 | | *yciN* | cytoplasmic protein | 1.21 | 1.97 |  |
| STM1734 | | *yciC* | hypothetical protein |  | 1.05 | 1.24 |
| STM1751 | | *hns* | DNA-binding protein H-NS |  | 1.61 | 1.05 |
| STM1822 | | *yoaB* | translation initiation inhibitor |  |  | 1.33 |
| STM1835 | | *rrmA* | 23S rRNA m1G745 methyltransferase | 1.10 |  |  |
| STM1837 | | *cspC* | cold shock-like protein |  | 1.40 |  |
| STM1838 | | *yobF* | cytoplasmic protein |  | 1.46 | 1.17 |
| STM1839 | | *-* | hypothetical protein |  | 1.66 | 1.26 |
| STM1840 | | *yobG* | PhoP/PhoQ regulator (MgrB) |  | 1.62 |  |
| STM1847 | | *yebR* | free methionine-(R)-sulfoxide reductase |  | 1.25 | 1.69 |
| STM1856 | | *-* | cytoplasmic protein |  | 1.45 | 1.21 |
| STM1862 | | *pagO* | integral membrane protein |  | 1.61 | 1.94 |
| STM1864 | | *-* | inner membrane protein |  |  | 2.33 |
| STM1880 | | *yebE* | inner membrane protein |  |  | 1.65 |
| STM1893 | | *znuB* | zinc ABC transporter permease |  | 1.28 |  |
| STM1907 | | *cutC* | copper homeostasis protein |  | 1.94 | 2.42 |
| STM1936 | | *yecH* | cytoplasmic protein |  | 2.20 | 1.96 |
| STM1941 | | *-* | inner membrane protein |  | 2.22 | 2.32 |
| STM1949 | | *yecF* | cytoplasmic protein |  |  | 1.54 |
| STM1964 | | *yedD* | lipoprotein |  | 1.49 | 1.61 |
| STM1965 | | *yedE* | inner membrane protein |  |  | 1.15 |
| STM1983 | | *dsrB* | protein | 1.31 | 1.15 |  |
| STM2008 | | *-* | periplasmic protein | 1.18 |  |  |
| **Gene** | **Name** | | **Description** | **Log_2_FC** | | |
| **Upregulated** | | |  | **Δ*rfaL*** | **Δ*rfaG*** | **Δ*rfaD*** |
| STM2098 | | *galF* | UTP--glucose-1-phosphate uridylyltransferase subunit |  | 1.28 |  |
| STM2157 | | *yehS* | cytoplasmic protein |  | 1.00 | 1.33 |
| STM2160 | | *yehV* | HTH-type transcriptional regulator (MlrA) |  | 1.14 |  |
| STM2281 | | *-* | LysR family transcriptional regulator |  | 1.23 |  |
| STM2183 | | *cdd* | cytidine deaminase |  |  | 1.43 |
| STM2211.S | | *yeiP* | elongation factor P |  |  | 1.40 |
| STM2309 | | *menD* | 2-succinyl-5-enolpyruvyl-6-hydroxy-3-cyclohexene-1-carboxylate synthase |  | 1.56 |  |
| STM2311 | | *elaB* | inner membrane protein |  | 1.02 |  |
| STM2357 | | *-* | amino acid transporter |  | 1.04 |  |
| STM2380 | | *yfcL* | cytoplasmic protein | 2.05 | 2.05 |  |
| STM2381 | | *yfcM* | cytoplasmic protein |  |  | 1.16 |
| STM2387 | | *sixA* | phosphohistidine phosphatase |  | 1.31 | 1.47 |
| STM2388 | | *fadJ* | multifunctional fatty acid oxidation complex subunit alpha |  | 1.35 |  |
| STM2400 | | *-* | inner membrane protein | 1.19 |  |  |
| STM2401 | | *ddg* | palmitoleoyl-ACP-dependent acyltransferase |  | 1.81 | 2.57 |
| STM2404 | | *-* | ion transport protein | 1.23 | 1.04 |  |
| STM2409 | | *nupC* | NUP family nucleoside transport protein |  |  | 1.05 |
| STM2413 | | *yfeC* | negative regulator |  | 1.02 |  |
| STM2414 | | *yfeD* | negative regulator |  | 1.35 |  |
| STM2429 | | *cysZ* | sulfate transport protein |  | 1.91 |  |
| STM2442 | | *cysW* | sulfate/thiosulfate ABC transporter permease |  |  | 2.00 |
| STM2470 | | *eutS* | carboxysome structural protein |  | 1.23 |  |
| STM2476 | | *ypfG* | periplasmic protein |  | 1.79 |  |
| STM2554 | | *hcaT* | 3-phenylpropionic acid transporter | 1.12 |  |  |
| STM2557 | | *cadC* | DNA-binding transcriptional activator |  | 1.73 |  |
| STM2560 | | *yjdL* | di-/tripeptide transport protein |  | 3.73 |  |
| STM2585A | | *-* | PagK-like protein |  | 1.95 |  |
| STM2599 | | *-* | transposase | 3.06 | 2.25 | 1.39 |
| STM2623 | | *-* | hypothetical protein |  | 1.26 |  |
| STM2638 | | *rseB* | anti-sigma E factor RseB |  | 1.39 | 1.29 |
| STM2639 | | *rseA* | anti-sigma E factor RseA |  |  | 1.41 |
| STM2640 | | *rpoE* | ECF RNA polymerase sigma factor |  | 1.87 | 2.46 |
| STM2663 | | *yfiO* | outer membrane protein assembly factor (BamD) |  |  | 1.49 |
| STM2672 | | *yfiN* | diguanylate cyclase/phosphodiesterase |  | 1.34 |  |
| STM2673 | | *rplS* | 50S ribosomal protein L19 |  |  | 1.23 |
| STM2674 | | *trmD* | tRNA (guanine-N(1)-)-methyltransferase |  |  | 1.63 |
| STM2675 | | *rimM* | ribosome maturation factor |  |  | 1.61 |
| **Gene** | **Name** | | **Description** | **Log_2_FC** | | |
| **Upregulated** | | |  | **Δ*rfaL*** | **Δ*rfaG*** | **Δ*rfaD*** |
| STM2676 | | *rpsP* | 30S ribosomal protein S16 |  |  | 1.14 |
| STM2700 | | *-* | phage tail fiber-like protein | 1.99 |  |  |
| STM2701 | | *-* | phage tail sheath-like protein | 2.01 |  |  |
| STM2703 | | *-* | hypothetical protein | 1.65 |  |  |
| STM2704 | | *-* | tail fiber assembly-like protein | 1.60 |  |  |
| STM2718 | | *-* | head completion-like protein | 2.52 |  |  |
| STM2720 | | *-* | major capsid-like protein | 1.80 |  |  |
| STM2721 | | *-* | capsid scaffold-like protein | 2.31 |  |  |
| STM2722 | | *-* | terminase-like protein | 1.82 |  |  |
| STM2723 | | *-* | portal vertex-like protein | 2.49 |  |  |
| STM2727 | | *-* | hypothetical protein | 1.29 |  |  |
| STM2728 | | *-* | hypothetical protein | 1.23 |  |  |
| STM2748 | | *-* | transcriptional regulator | 1.16 |  |  |
| STM2781 | | *virK* | VirK-like protein |  |  | 1.77 |
| STM2782 | | *mig-14* | mig-14; transcriptional activator |  |  | 1.91 |
| STM2783 | | *nixA* | nickel transporter |  |  | 1.42 |
| STM2786 | | *-* | tricarboxylic transport protein |  |  | 2.72 |
| STM2788 | | *-* | tricarboxylic transport protein |  |  | 2.10 |
| STM2791 | | *gabD* | succinate-semialdehyde dehydrogenase I | 1.73 | 1.61 | 1.55 |
| STM2799 | | *stpA* | DNA binding protein |  | 1.06 | 1.28 |
| STM2801 | | *ygaC* | cytoplasmic protein |  | 1.56 |  |
| STM2813 | | *emrR* | negative regulator of the multidrug operon emrRAB |  | 1.07 | 1.06 |
| STM2815 | | *emrB* | multidrug transport protein |  | 1.34 |  |
| STM2844 | | *-* | hypothetical protein | 1.84 |  |  |
| STM2857 | | *hypD* | hydrogenase expression/formation protein |  | 1.05 |  |
| STM2864 | | *sitD* | Fur regulated iron ABC transporter permease | 2.31 | 2.04 | 1.58 |
| STM2920 | | *-* | transcriptional regulator | 1.18 |  |  |
| STM2931 | | *ftsB* | cell division protein |  |  | 1.11 |
| STM2958 | | *barA* | sensory histidine kinase |  | 1.17 |  |
| STM2986.Sc | | *-* | integral membrane protein |  | 1.44 |  |
| STM3007 | | *ygdR* | POT family peptide transport protein |  | 1.35 |  |
| STM3013 | | *lysA* | diaminopimelate decarboxylase | 1.52 |  |  |
| STM3030 | | *-* | periplasmic protein |  |  | 1.70 |
| STM3031 | | *-* | Ail/OmpX-like protein |  |  | 2.26 |
| STM3036 | | *-* | inner membrane protein |  | 2.02 | 2.07 |
| STM3060 | | *ygfE* | Z-ring-associated protein | 1.82 | 2.19 | 1.39 |
| STM3063 | | *rpiA* | ribose-5-phosphate isomerase A |  | 1.03 | 1.00 |
| STM3065 | | *yggE* | oxidative stress defense protein |  | 1.49 |  |
| **Gene** | **Name** | | **Description** | **Log_2_FC** | | |
| **Upregulated** | | |  | **Δ*rfaL*** | **Δ*rfaG*** | **Δ*rfaD*** |
| STM3066 | | *yggA* | arginine exporter protein (ArgO) |  | 1.30 |  |
| STM3073 | | *-* | cobalt ABC transporter permease |  |  | 1.11 |
| STM3091 | | *galP* | galactose/proton symporter |  | 1.37 |  |
| STM3096 | | *yqgE* | hypothetical protein |  | 1.14 |  |
| STM3105 | | *yggM* | periplasmic protein |  | 1.49 |  |
| STM3107 | | *yggN* | periplasmic protein |  |  | 1.93 |
| STM3132 | | *-* | xylanase/chitin deacetylase |  | 1.40 |  |
| STM3133 | | *-* | amidohydrolase |  | 1.66 |  |
| STM3143 | | *hybG* | hydrogenase 2 accessory protein | 1.03 | 1.10 |  |
| STM3158 | | *exbD* | biopolymer transport protein |  | 1.01 | 1.18 |
| STM3162 | | *yghB* | inner membrane protein |  | 1.43 |  |
| STM3166.S | | *-* | cation transporter | 1.71 | 2.09 |  |
| STM3167 | | *-* | diadenosine tetraphosphate hydrolase |  |  | 1.01 |
| STM3189 | | *ygiD* | cytoplasmic protein | 1.35 | 1.44 | 1.90 |
| STM3197 | | *glgS* | glycogen synthesis protein | 1.36 |  |  |
| STM3203 | | *ygiM* | SH3 domain-containing protein |  | 1.30 | 1.34 |
| STM3227 | | *yqjB* | modulator protein (MzrA) |  | 1.02 |  |
| STM3245 | | *tdcA* | DNA-binding transcriptional activator |  | 2.45 |  |
| STM3257 | | *-* | D-tagatose-1,6-bisphosphate aldolase subunit (GatZ) |  | 1.71 | 1.09 |
| STM3274 | | *yhbU* | protease |  |  | 1.12 |
| STM3281 | | *nlpI* | lipoprotein | 1.05 |  |  |
| STM3284 | | *truB* | tRNA pseudouridine synthase B |  |  | 1.06 |
| STM3298.S | | *yhbY* | RNA-binding protein | 1.06 | 1.78 | 1.74 |
| STM3299 | | *greA* | transcription elongation factor |  | 1.02 | 1.40 |
| STM3309 | | *yrbB* | STAS domain-containing protein | 1.06 |  |  |
| STM3339 | | *nanA* | N-acetylneuraminate lyase |  |  | 1.27 |
| STM3344 | | *rpsI* | 30S ribosomal protein S9 |  | 1.49 | 3.01 |
| STM3345 | | *rplM* | 50S ribosomal protein L13 |  |  | 1.28 |
| STM3349 | | *degS* | serine endoprotease |  | 1.10 |  |
| STM3352 | | *oadA* | oxaloacetate decarboxylase subunit alpha |  |  | 2.52 |
| STM3372 | | *mreD* | rod shape-determining protein |  | 1.18 |  |
| STM3376 | | *yhdH* | oxidoreductase |  | 1.06 | 1.70 |
| STM3392 | | *yhdV* | outer membrane lipoprotein |  | 2.39 |  |
| STM3414 | | *rplQ* | 50S ribosomal protein L17 |  |  | 1.73 |
| STM3415 | | *rpoA* | DNA-directed RNA polymerase subunit alpha |  |  | 1.55 |
| STM3416 | | *rpsD* | 30S ribosomal protein S4 |  |  | 1.15 |
| STM3417 | | *rpsK* | 30S ribosomal protein S11 |  |  | 1.05 |
| STM3420 | | *secY* | preprotein translocase subunit |  |  | 1.27 |
| STM3431 | | *rpsQ* | 30S ribosomal protein S17 |  |  | 1.52 |
| **Gene** | **Name** | | **Description** | **Log_2_FC** | | |
| **Upregulated** | | |  | **Δ*rfaL*** | **Δ*rfaG*** | **Δ*rfaD*** |
| STM3432 | | *rpmC* | 50S ribosomal protein L29 |  |  | 2.39 |
| STM3433 | | *rplP* | 50S ribosomal protein L16 |  |  | 1.95 |
| STM3434 | | *rpsC* | 30S ribosomal protein S3 |  |  | 2.13 |
| STM3435 | | *rplV* | 50S ribosomal protein L22 |  |  | 1.65 |
| STM3436 | | *rpsS* | 30S ribosomal protein S19 |  |  | 1.76 |
| STM3437 | | *rplB* | 50S ribosomal protein L2 |  |  | 1.67 |
| STM3438 | | *rplW* | 50S ribosomal protein L23 |  |  | 1.37 |
| STM3439 | | *rplD* | 50S ribosomal protein L4 |  |  | 1.32 |
| STM3440 | | *rplC* | 50S ribosomal protein L3 |  |  | 1.51 |
| STM3441 | | *rpsJ* | 30S ribosomal protein S10 |  |  | 1.79 |
| STM3453 | | *fkpA* | FKBP-type peptidyl-prolyl cis-trans isomerase |  | 1.18 | 2.06 |
| STM3501 | | *envZ* | osmolarity sensor protein |  | 1.02 |  |
| STM3513 | | *malQ* | 4-alpha-glucanotransferase |  | 2.22 | 2.41 |
| STM3514 | | *malP* | maltodextrin phosphorylase |  | 2.93 | 2.59 |
| STM3539 | | *asd* | aspartate-semialdehyde dehydrogenase |  | 1.26 |  |
| STM3568 | | *rpoH* | RNA polymerase sigma factor |  | 1.21 | 1.21 |
| STM3578 | | *yhhP* | tRNA 2-thiouridine synthesizing protein (TusA) |  | 1.63 |  |
| STM3580 | | *-* | inner membrane lipoprotein |  | 1.15 |  |
| STM3635 | | *yhjW* | phosphoethanolamine transferase |  | 1.07 |  |
| STM3645 | | *yiaD* | outer membrane lipoprotein |  |  | 1.57 |
| STM3648 | | *yiaG* | transcriptional regulator |  | 1.34 |  |
| STM3663 | | *bax* | hypothetical protein |  | 1.04 |  |
| STM3664 | | *malS* | alpha-amylase |  | 3.85 | 2.85 |
| STM3665 | | *avtA* | valine--pyruvate transaminase |  | 1.69 |  |
| STM3666 | | *ysaA* | oxidoreductase |  | 1.09 |  |
| STM3681 | | *-* | transcriptional regulator |  |  | 1.29 |
| STM3712 | | *rfaC* | ADP-heptose--LPS heptosyltransferase | 3.25 | 1.15 |  |
| STM3729 | | *radC* | hypothetical protein |  | 1.43 | 1.80 |
| STM3744 | | *recG* | ATP-dependent DNA helicase |  |  | 1.27 |
| STM3761 | | *slsA* | inner membrane protein |  | 2.02 | 1.62 |
| STM3763 | | *mgtB* | magnesium-transporting ATPase |  |  | 1.93 |
| STM3765 | | *yicL* | inner membrane transporter |  | 1.19 |  |
| STM3787 | | *uhpT* | hexose phosphate transport protein | 1.24 | 2.32 | 2.45 |
| STM3797 | | *ivbL* | ilvB operon leader peptide | 2.64 | 2.70 | 2.41 |
| STM3803 | | *yidF* | cytoplasmic protein |  | 1.55 |  |
| STM3804 | | *yidG* | inner membrane protein | 1.26 | 1.68 |  |
| STM3810 | | *yidQ* | outer membrane lipoprotein |  | 1.31 |  |
| STM3825 | | *torT* | periplasmic sensor |  | 1.33 | 1.14 |
| STM3826 | | *torS* | sensory kinase |  | 1.30 |  |
| STM3855 | | *pstA* | phosphate ABC transporter permease subunit |  |  | 1.43 |
| **Gene** | **Name** | | **Description** | **Log_2_FC** | | |
| **Upregulated** | | |  | **Δ*rfaL*** | **Δ*rfaG*** | **Δ*rfaD*** |
| STM3872 | | *atpI* | F0F1 ATP synthase subunit I |  | 1.97 | 1.43 |
| STM3878.S | | *yieM* | protein (ViaA) |  | 1.04 |  |
| STM3884 | | *rbsB* | D-ribose ABC transporter substrate-binding protein | 1.08 |  |  |
| STM3900 | | *ilvL* | ilvG operon leader peptide | 2.91 |  |  |
| STM3910 | | *ppiC* | peptidyl-prolyl cis-trans isomerase C |  | 1.08 | 1.14 |
| STM3913 | | *gppA* | guanosine-5'-triphosphate,3'-diphosphate pyrophosphatase | 1.22 | 1.40 |  |
| STM3914 | | *rhlB* | ATP-dependent RNA helicase |  | 1.39 |  |
| STM3915 | | *trxA* | thioredoxin |  |  | 1.06 |
| STM3926 | | *wzxE* | O-antigen translocase |  | 1.14 |  |
| STM3975 | | *tatC* | Sec-independent protein translocase protein |  | 1.51 | 1.27 |
| STM3985 | | *yigZ* | cytoplasmic protein | 1.03 |  |  |
| STM3998 | | *yihG* | acyltransferase | 2.09 | 2.08 | 1.12 |
| STM4002 | | *-* | cytoplasmic protein | 1.33 |  |  |
| STM4028 | | *yihZ* | D-tyrosyl-tRNA(Tyr) deacylase |  |  | 1.26 |
| STM4049 | | *rhaR* | HTH-type transcriptional activator |  | 1.19 |  |
| STM4056.S | | *yiiM* | hypothetical protein |  |  | 1.12 |
| STM4057 | | *-* | inner membrane protein |  | 1.33 | 1.38 |
| STM4060 | | *cpxP* | cpx regulon periplasmic repressor |  | 1.79 | 2.76 |
| STM4089 | | *menG* | ribonuclease activity regulator protein (RraA) |  | 1.60 |  |
| STM4098 | | *-* | arylsulfate sulfotransferase | 1.42 | 1.36 |  |
| STM4128 | | *yijD* | inner membrane protein |  | 1.04 |  |
| STM4131 | | *murI* | glutamate racemase |  | 1.05 |  |
| STM4147 | | *secE* | preprotein translocase subunit |  | 1.05 | 1.09 |
| STM4149 | | *rplK* | 50S ribosomal protein L11 |  |  | 1.12 |
| STM4150 | | *rplA* | 50S ribosomal protein L1 |  |  | 1.29 |
| STM4151 | | *rplJ* | 50S ribosomal protein L10 |  |  | 2.01 |
| STM4152 | | *rplL* | 50S ribosomal protein L7/L12 |  |  | 1.91 |
| STM4191 | | *-* | cytoplasmic protein |  | 1.80 |  |
| STM4196 | | *-* | cytoplasmic protein |  | 1.06 |  |
| STM4222.S | | *yjbE* | outer membrane protein |  | 5.09 | 5.32 |
| STM4243 | | *yjbN* | tRNA-dihydrouridine synthase A | 1.06 |  |  |
| STM4247 | | *alr* | alanine racemase | 1.46 |  |  |
| STM4276 | | *-* | cytoplasmic protein | 1.45 |  |  |
| STM4283 | | *gltP* | glutamate/aspartate:proton symporter | 1.44 | 1.14 |  |
| STM4284 | | *yjcO* | hypothetical protein |  | 1.19 |  |
| STM4287.S | | *phnO* | aminoalkylphosphonic acid N-acetyltransferase |  |  | 1.22 |
| STM4289 | | *phnA* | hypothetical protein |  |  | 1.42 |
| STM4290 | | *proP* | proline/betaine transporter |  | 1.02 |  |
| **Gene** | **Name** | | **Description** | **Log_2_FC** | | |
| **Upregulated** | | |  | **Δ*rfaL*** | **Δ*rfaG*** | **Δ*rfaD*** |
| STM4302 | | *-* | cytoplasmic protein |  | 1.28 |  |
| STM4304 | | *dcuS* | sensory histidine kinase DcuS |  | 1.16 |  |
| STM4320 | | *-* | MerR family regulatory protein |  | 1.11 |  |
| STM4328 | | *yjeH* | transporter |  | 1.13 |  |
| STM4332 | | *yjeJ* | inner membrane protein |  | 1.79 |  |
| STM4336 | | *ecnB* | entericidin B | 1.24 |  |  |
| STM4345 | | *yjeM* | inner membrane transporter |  | 2.12 |  |
| STM4347 | | *yjeP* | mechanosensitive channel protein |  |  | 1.40 |
| STM4360 | | *miaA* | tRNA dimethylallyltransferase |  |  | 1.06 |
| STM4390 | | *-* | cytoplasmic protein |  |  | 1.56 |
| STM4391 | | *rpsF* | 30S ribosomal protein S6 |  |  | 1.49 |
| STM4392 | | *priB* | primosomal replication protein N |  |  | 1.49 |
| STM4393 | | *rpsR* | 30S ribosomal protein S18 |  |  | 1.18 |
| STM4394 | | *rplI* | 50S ribosomal protein L9 |  |  | 1.15 |
| STM4397 | | *fklB* | peptidyl-prolyl cis-trans isomerase |  |  | 1.72 |
| STM4406.S | | *ytfK* | hypothetical protein | 1.63 | 1.09 |  |
| STM4412 | | *-* | pemease |  | 2.44 |  |
| STM4414 | | *ppa* | inorganic pyrophosphatase |  |  | 1.77 |
| STM4451 | | *nrdG* | anaerobic ribonucleotide-triphosphate reductase-activating protein |  | 1.42 | 1.10 |
| STM4452 | | *nrdD* | anaerobic ribonucleoside-triphosphate reductase |  | 1.12 |  |
| STM4482 | | *idnT* | GntP family L-idonate transport protein | 1.34 |  |  |
| STM4486 | | *yjgB* | alcohol dehydrogenase |  |  | 1.29 |
| STM4504 | | *-* | cytoplasmic protein |  | 1.36 |  |
| STM4508.1N | | *-* | hypothetical protein |  | 2.30 |  |
| STM4521 | | *yjiS* | cytoplasmic protein |  |  | 999.00 |
| STM4532 | | *yjiY* | carbon starvation protein |  | 1.38 |  |
| STM4538 | | *-* | PTS system mannose-specific transporter subunit IID | 1.48 |  |  |
| STM4546 | | *yjjP* | membrane protein |  | 1.57 |  |
| STM4549 | | *-* | cytoplasmic protein |  |  | 1.08 |
| STM4552 | | *-* | inner membrane protein |  | 1.58 |  |
| STM4565 | | *yjjW* | pyruvate formate lyase-activating enzyme |  | 1.20 |  |
| STM4571 | | *-* | outer membrane protein |  | 1.65 |  |
| STM4578 | | *serB* | 3-phosphoserine phosphatase |  |  | 1.12 |
