# Supplementary material for: Regulation of Flagellum Biosynthesis in Response to Cell Envelope Stress in Salmonella enterica Serovar Typhimurium
Source: mBio. 2018 May 1;9(3):e00736-17. doi: 10.1128/mBio.00736-17 (PMC5930307; doi:10.1128/mBio.00736-17)
Supplement: TABLE S3 [file mbo002183865st3.docx]

**Table S3:** ***Salmonella enterica* serovar Typhimurium strains**

| **Strain** | **Relevant characteristics** | **Reference or source** |
| --- | --- | --- |
| TH437 | LT2 | J. Roth |
| TH13865 | Δ*flhDC*::FKF | Lab collection |
| EM3014 | Δ*rflP* | Lab collection |
| EM3016 | Δ*rfaL*::FKF | This study |
| EM3017 | Δ*rflP* Δ*rfaL*::FKF | This study |
| EM3092 | Δ*rfaG*:*aph* | (Frahm *et al.*, 2015) |
| EM3093 | Δ*rflP* Δ*rfaG*::*aph* | This study |
| EM3094 | Δ*rfaD*::*aph* | (Frahm *et al.*, 2015) |
| EM3095 | Δ*rflP* Δ*rfaD*::*aph* | This study |
| TH5861 | Δ*hin*5717::FCF (fliC-ON) | Lab collection |
| EM4764 | Δ*hin*5717::FCF (fliC-ON) Δ*rflP*::*tetRA* | This study |
| EM4927 | Δ*hin*5717::FCF (fliC-ON) Δ*rfaL*::FKF | This study |
| EM4928 | Δ*hin*5717::FCF (fliC-ON) Δ*rflP*::*tetRA* Δ*rfaL*::FKF | This study |
| EM4762 | Δ*hin*5717::FCF (fliC-ON) Δ*rfaG*::*aph* | This study |
| EM4929 | Δ*hin*5717::FCF (fliC-ON) Δ*rflP*::*tetRA* Δ*rfaG*::*aph* | This study |
| EM4763 | Δ*hin*5717::FCF (fliC-ON) Δ*rfaD*::*aph* | This study |
| EM4930 | Δ*hin*5717::FCF (fliC-ON) Δ*rflP*::*tetRA* Δ*rfaD*::*aph* | This study |
| EM4410 | Δ*araBAD*::*rfaG* Δ*rfaG*::*aph* | Lab collection |
| EM4411 | Δ*araBAD*::*rfaD* Δ*rfaD*::*aph* | Lab collection |
| TH2089 | *fliL*5100::Mu*d*J | Lab collection |
| TH2775 | *flhC*5213::Mu*d*J | Lab collection |
| TH3933 | *motA*5461::Mu*d*J | Lab collection |
| TH7365 | *fljB*5001::Mu*d*J Δ*hin*5718::FRT | Lab collection |
| EM3170 | *fliL*5100::Mu*d*J (kanamycin in Mu*d*J replaced by FCF) | This study |
| EM3171 | *flhC*5213::Mu*d*J (kanamycin in Mu*d*J replaced by FCF) | This study |
| EM3172 | *motA*5461::Mu*d*J (kanamycin in Mu*d*J replaced by FCF) | This study |
| EM3173 | *fljB*5001::Mu*d*J Δ*hin*5718::FRT (kanamycin in Mu*d*J replaced by FCF) | This study |
| EM4754 | *fliL*5100::Mu*d*J (kanamycin in Mu*d*J replaced by FCF) Δ*rfaG*::*aph* | This study |
| EM4755 | *flhC*5213::Mu*d*J (kanamycin in Mu*d*J replaced by FCF) Δ*rfaG*::*aph* | This study |
| EM4756 | *motA*5461::Mu*d*J (kanamycin in Mu*d*J replaced by FCF) Δ*rfaG*::*aph* | This study |
| EM4757 | *fljB*5001::Mu*d*J Δ*hin*5717::Frt (kanamycin in Mu*d*J replaced by FCF) Δ*rfaG*::*aph* | This study |
| EM4758 | *fliL*5100::Mu*d*J (kanamycin in Mu*d*J replaced by FCF) Δ*rfaD*::*aph* | This study |
| EM4759 | *flhC*5213::Mu*d*J (kanamycin in Mu*d*J replaced by FCF) Δ*rfaD*::*aph* | This study |
| EM4760 | *motA*5461::Mu*d*J (kanamycin in Mu*d*J replaced by FCF) Δ*rfaD*::*aph* | This study |
| **Strain** | **Relevant characteristics** | **Reference or source** |
| EM4761 | *fljB*5001::Mu*d*J Δ*hin*5717::Frt (kanamycin in Mu*d*J replaced by FCF) Δ*rfaD*::*aph* | This study |
| EM3731 | *fliL*5100::Mu*d*J (kanamycin in Mu*d*J replaced by Frt) Δ*rfaG*::*tetRA* | This study |
| TH13863 | Δ*rflP*::Frt-*lac*-*km* | Lab collection |
| EM4917 | Δ*cpx*::FCF Δ*rflP*::Frt-*lac*-*km* | This study |
| EM4918 | Δ*cpxP*::FCF Δ*rflP*::Frt-*lac*-*km* | This study |
| EM4919 | Δ*rpoE*::FCF Δ*rflP*::Frt-*lac*-*km* | This study |
| EM4920 | Δ*nlpC*::FCF Δ*rflP*::Frt-*lac*-*km* | This study |
| EM4921 | Δ*yjbE*::FCF Δ*rflP*::Frt-*lac*-*km* | This study |
| EM4922 | Δ*rstA*::FCF Δ*rflP*::Frt-*lac*-*km* | This study |
| EM4923 | Δ*rstB*::FCF Δ*rflP*::Frt-*lac*-*km* | This study |
| EM4924 | Δ*htrA*::FCF Δ*rflP*::Frt-*lac*-*km* | This study |
| EM4985 | Δ*cpxP*::Frt Δ*rstB*::FCF Δ*rflP*::Frt-*lac*-*km* | This study |
| EM1438 | *flhC*22735::3FLAG-FRT | Lab collection |
| EM6198 | *flhC*22735::3FLAG-FRT Δ*rfaG*::*aph* | This study |
