# Supplementary material for: Regulation of Flagellum Biosynthesis in Response to Cell Envelope Stress in Salmonella enterica Serovar Typhimurium
Source: mBio. 2018 May 1;9(3):e00736-17. doi: 10.1128/mBio.00736-17 (PMC5930307; doi:10.1128/mBio.00736-17)
Supplement: TABLE S4 [file mbo002183865st4.docx]

**Table S4: Oligonucleotides used in this study**

| **Name** | **5'-3' sequence** |
| --- | --- |
| 5'-rflP_qPCR_fw | TCATGCACGCTATTGTTGCC |
| 3'-rflP_qPCR_rv | AATGCGCTAAACCCCAATGG |
| 5'-rpoE_qPCR-fw | AGCAGTTAACGGACCAGGTC |
| 3'-rpoE_qPCR-rv | AAGATACGTGAACGCACCGT |
| 5'-wzzB_qPCR_fw | CGATCCGGAACAGATTGATT |
| 3'-wzzB_qPCR_rv | AGGTAGCCTACAGCCAGCAA |
| gyrB_qPCR_new-fw | ACGCTCTGTCGCAAAAACTG |
| gyrB_qPCR_new-rv | ACCATCGTGCCGGTTTTATC |
| gmk_qPCR_new-fw | TTTTGCCGCCGTCAAAGATC |
| gmk_qPCR_new-rv | ATGGCTCATTTCTGCAACCG |
| rpoD_qPCR_new-fw | ACACCATCAAAGCGAAAGGC |
| rpoD_qPCR_new-rv | TCATCACGCGCATACTGTTG |
| flhDC-qPCR-fw | GTAGGCAGCTTTGCGTGTAG |
| flhDC-qPCR-rv | TCCAGCAGTTGTGGAATAATATCG |
| flgE-qPCR-fw | AACGTCTATTTTGTGAAAACCAAAG |
| flgE-qPCR-rv | AGACTCCAGAATCCCGTTTTC |
| 5'-fljB_qPCR-60C-fw | TACACCGGCAGTTGTTTCAG |
| 3'-fljB_qPCR-60C-rv | CCGCCTTCAATTGTCTTACC |
| 5'-fliC_qPCR-60C-fw | TGATAAGACGAACGGTGAGG |
| 3'-fliC_qPCR-60C-rv | AACACCTGCTGCTGTCAATG |
| 5'-flagellin_qPCR-60C-fw | AACGACGGTATCTCCATTGC |
| 3'-flagellin_qPCR-60C-rv | ATTTCAGCCTGGATGGAGTC |
| 5'_DcpxP_FCF_fw | ACAGAAAAGATTTTGGGAGCAAGCGATGCGCAAAGTTACCccatatgaatatcctcctta |
| 3'_DcpxP_FCF_rv | GTCTATGGCAAGGAAAACAGGGTTGTTACTGGGAACGTGAgtgtaggctggagctgcttc |
| 5'_Dcpx_locus_FCF_fw | GCCTGCATTCGCAGGCCGATGGTTTTTAGGTTCGCTTGTAccatatgaatatcctcctta |
| 5'_DhtrA_FCF_fw | TACCTGTTAATCGAGATTGAAACACATGAAAAAAACCACAccatatgaatatcctcctta |
| 3'_DhtrA_FCF_rv | CATGGCGGAAGGGGGACAAAGGTGATTACTGCATCAGCAAgtgtaggctggagctgcttc |
| 5'_DnlpC_FCF_fw | AATAAAAACAGAGGATTGTTGCGGCATGCGTTTTTGGCTTccatatgaatatcctcctta |
| 3'_DnlpC_FCF_rv | TATAAAATTTATATCGTCTGCGAGGTTAAATTCGCCGTGCgtgtaggctggagctgcttc |
| 3'_DrpoE_FCF_rv | TACCTTTTCCAGTATCCCGCTATCGTCAACGCCTGATAAGgtgtaggctggagctgcttc |
| 5'_DrpoE_FCF_fw | TTGGTTTGGGGAGACATTACCTCGGATGAGCGAGCAGTTAccatatgaatatcctcctta |
| 5'_DrstA_FCF_fw | GCGTTTTCTATTCTCCATTTATAATATGAACCGCATTGTAccatatgaatatcctcctta |
| 3'_DrstA_FCF_rv | AGGCAGGTAAAATCTGTCGGCTAACTTATCCCGTCGTTTCgtgtaggctggagctgcttc |
| **Name** | **5'-3' sequence** |
| 5'_DrstB_FCF_fw | ATGTATTGAGATCCGGTGGGCGTTGATGAAAAAGCTGTTTccatatgaatatcctcctta |
| 3'_DrstB_FCF_rv | CCGCTCAACCAGCGTGCAAAATGCGTCAGGCAGCGGTCATgtgtaggctggagctgcttc |
| 3'_DyjbE_FCF_rv | GTGGTTATGGTTAAACGTACCGATACTACTGGGTACTGGTgtgtaggctggagctgcttc |
| 5'_DyjbE_FCF_fw | ATTCATTCAATGAAGGGAAGTTATGATGAAAAAAGTACTGccatatgaatatcctcctta |
